# Adjuvant combination and antigen multimerization shape neutralizing antibody and T cell responses to a SARS-CoV-2 RBD subunit vaccine

**DOI:** 10.3389/fimmu.2025.1610422

**Published:** 2025-07-17

**Authors:** João Pedro da Silva Nunes, Mariângela de Oliveira Silva, Juliana de Souza Apostolico, Isabela Pazotti Daher, Rodolfo Ferreira Marques, Marcio Massao Yamamoto, Alexia Adrianne Venceslau Brito Carvalho, Maria Fernanda de Castro-Amarante, Edison Luiz Durigon, Carsten Wrenger, Luiz Mario Ramos Janini, Edmarcia Elisa de Souza, Robert Andreata-Santos, Juliana Terzi Maricato, Edecio Cunha-Neto, Jorge Kalil, Silvia Beatriz Boscardin, Daniela Santoro Rosa

**Affiliations:** ^1^ Departamento de Microbiologia, Imunologia e Parasitologia, Universidade Federal de São Paulo-Escola Paulista de Medicina (UNIFESP/EPM), São Paulo, Brazil; ^2^ Departamento de Parasitologia, Universidade de São Paulo (USP), São Paulo, Brazil; ^3^ Laboratório de Imunologia, Instituto do Coração (InCor), Hospital das Clínicas- Faculdade de Medicina- Universidade de São Paulo (HCFMUSP), São Paulo, Brazil; ^4^ Departamento de Microbiologia, Universidade de São Paulo (USP), São Paulo, Brazil; ^5^ Laboratório de Investigação Médica (LIM-19), Faculdade de Medicina- Universidade de São Paulo (FMUSP), São Paulo, Brazil; ^6^ Instituto de Investigação em Imunologia (iii), Instituto Nacional de Ciência e Tecnologia (INCT), São Paulo, Brazil

**Keywords:** SARS-CoV-2, RBD, vaccine, recombinant protein, adjuvant

## Abstract

**Introduction:**

The rapid development and deployment of multiple safe and effective COVID-19 vaccines were critical cornerstones of pandemic control. However, vaccine inequity and the emergence of new variants of concern (VOCs) highlighted major gaps in the global strategy to control SARS-CoV-2 infection. Despite the use of distinct platforms, most approved vaccines utilize the Spike protein as the main antigen due to its pivotal role in virus entry, mediated by the receptor binding domain (RBD). In this context, RBD stands out as a promising antigen for a subunit vaccine candidate, as it is the main target of neutralizing antibodies, has a well-established scalable production pipeline, and has proven safety. Approaches to enhance RBD immunogenicity encompass the addition of adjuvants and antigen multimerization.

**Methods:**

In this study, we compared the immunogenic properties of the Wuhan RBD monomer and homodimer with an RBD heterotrimer formulation composed of the Delta, Beta and Gamma variants. We also screened different adjuvants to optimize both humoral and cellular immunity.

**Results:**

Our results showed that immunization with the RBD dimer and trimer, in the presence of the adjuvant AddaS03, elicited a higher humoral response and a broader neutralization profile. Additionally, RBD-trimer immunization more efficiently inhibited viral replication in the lungs of mice challenged with the ancestral Wuhan strain compared to the monomer. We further optimized our vaccine formulation by combining the adjuvants AddaS03 and Poly I:C, which demonstrated a synergistic effect, integrating the potent humoral response induced by AddaS03 with the cellular Th1 skewing capacity of Poly I:C. The AddaS03+ Poly I:C mixture induced antibodies with higher affinity and an increased frequency of RBD-specific IgG2c-producing bone marrow plasma cells, highlighting the potential of this adjuvant combination to generate long-lived memory plasma cells. Additionally, we identified sequences within the RBD that induced specific IFNγ T cell responses. Peptide 12 (393-TNVYADSFVIRGDEVRQ-409) emerged as the immunodominant CD4 T cell epitope, whereas peptides 28 (505-YQPYRVVVLSFELLHAP-521) and 29 (512-VLSFELLHAPATVCGPK-528) successfully activated CD8 T cells.

**Conclusions:**

These findings underscore that antigen multimerization and the strategic combination of adjuvants can significantly improve vaccine immunogenicity.

## Introduction

Vaccination has consistently proven to be the most effective strategy for preventing infectious diseases. One of the greatest scientific achievements of the COVID-19 pandemic was the rapid development and deployment of multiple safe and effective vaccines. However, the unequal global distribution of vaccines, inconsistent public health measures, and the emergence of new variants of concern (VOCs) (1) have highlighted substantial gaps in controlling SARS-CoV-2 infection (2).

In response to these challenges, subunit vaccines have emerged as a promising alternative for producing safe and affordable vaccines, particularly for low- and middle-income countries (3), facilitating global production and distribution. In this scenario, recombinant protein–based subunit vaccines offer several advantages as they rely on well-established technologies, typically do not require ultra-low temperature (-60°C to -80°C) storage, with most being stable at 2°C –8°C, and have demonstrated safety and efficacy across various age groups (4).

The available COVID-19 vaccines vary greatly among platforms, but the vast majority of these vaccines utilize the Spike (S) glycoprotein as the main antigen (5). S-specific neutralizing antibodies (nAbs) strongly correlate with protection against infection, and the receptor-binding domain (RBD) which is essential for receptor docking, has emerged as the main target of these antibodies (6, 7). Thus, the development of RBD-based subunit vaccines (8) represents a compelling alternative, as these vaccines target major neutralizing domains. Despite the potential of RBD-based subunit vaccines, there are considerable challenges to overcome, particularly their limited ability to stimulate a robust immune response due to small molecular size (9, 10). Enhancing RBD immunogenicity may require strategies such as the addition of adjuvants and antigen multimerization (11, 12).

Adjuvants enhance the magnitude, quality, and durability of vaccine-induced immune responses by activating the innate immune system through different mechanisms (13). These include, but are not limited to: i) specifically targeting cells such as monocytes, dendritic cells (DCs), macrophages, and neutrophils through pattern recognition receptors (PRRs), including Toll-like Receptors (TLRs; e.g., Poly I:C or CpG, synthetic compounds that mimic nucleic acids and activate TLR3 and TLR9, respectively) (13); ii) TLR-independent MyD88 activation (e.g., MF59 and AS03, oil-in-water emulsion adjuvants) (14, 15), iii) inflammasome activation (e.g., Alhydrogel, an aluminum salt that activates NLRP3) (16) and other mechanisms that are critical to immune activation. Adjuvants can also modulate the pharmacokinetics of the vaccine antigen, thereby optimizing the generation of specific immune responses (17). The nature of the adjuvant and its mechanism of action directly impacts its efficacy, particularly in protein-based subunit vaccines. For instance, some compounds, such as Alhydrogel, predominantly induce Th2-biased responses, while others such as Poly I:C and CpG ODN, promote skewed Th1 responses (18, 19).

Previous studies have highlighted the superiority of RBD multimerization (20–25). This effect may be attributed to the multimeric assembly of the protein, which can enhance its interaction with the B cell receptor (BCR), promote BCR clustering in lipid rafts, and thereby potentiate signal transduction in B cells (26). Multimers might also expose distinct and more avid conformational sites for antibody recognition, thereby optimizing the neutralization capacity of these antibodies. Additionally, protein processing and the presentation of class I and class II epitopes can concomitantly play a crucial role, as multimers present a higher quantity of such sequences, and T cell help is essential for efficient B cell activation in the context of protein-based vaccines (27).

Moreover, the reduced efficacy of vaccines based on the ancestral Wuhan Spike against VOCs is well-documented (28). To ensure proper efficacy and circumvent virus mutations, vaccine boosters containing antigens from circulating VOCs, including Spikevax (Moderna), Comirnaty (Pfizer-BioNTech), and the protein-adjuvanted Nuvaxovid (Novavax) (29, 30) have been used. In this context, the effectiveness of the ancestral Wuhan strain RBD against VOCs and the implications of introducing mutated antigens into RBD-based vaccine formulations have emerged as important areas of investigation.

Here, we assessed the humoral and cellular immune responses elicited by recombinant monomer and tandem homodimer of ancestral Wuhan RBD proteins, as well as a tandem heterotrimer RBD composed of Delta, Beta and Gamma variants, in a murine model. Our findings demonstrated that immunization with dimer and trimer generated higher and broader neutralization of VOCs, with the RBD heterotrimer being particularly effective against Omicron variants. Additionally, immunization with the dimer and trimer efficiently controlled SARS-CoV-2 replication in lung tissue, although all three proteins were effective in protecting mice from death against viral challenge. Furthermore, the combination of the adjuvants AddaS03 and Poly I:C promoted a synergistic effect, integrating the potent humoral response induced by AddaS03 with the cellular Th1- skewing capacity of Poly I:C.

## Materials and methods

### Constructs and protein expression

The monomeric RBD protein from the first isolate, Wuhan-Hu-1 (GenBank: MN908947.3), was produced from a plasmid kindly provided by Dr. Florian Krammer (Icahn School of Medicine at Mount Sinai, USA). Briefly, the RBD gene sequence (amino acids 319–541) was cloned between the Spike signal peptide (MFVFLVLLPLVSSQ) and a hexahistidine tag into the mammalian expression vector pCAGGS (31).

To generate the RBD dimer protein, two Wuhan-Hu-1 RBD monomer sequences (amino acids 319-537) were cloned in tandem as previously described (11). The tandem RBD sequences were codon-optimized for mammalian expression and cloned between the IgE signal peptide (MDWTWILFLVAAATRVHS) and a hexahistidine tag into the mammalian expression vector pcDNA3.1 by GenScript (Piscataway, NJ, USA).

To generate the RBD-trimer protein, three RBD monomeric sequences (amino acids 319-537) from the Delta (mutations L452R and T458K), Beta (mutations K417N, E484K and N501Y) and Gamma (mutations K417T, E484K and N501Y) variants were codon-optimized for mammalian expression and cloned between the IgE signal peptide (MDWTWILFLVAAATRVHS) and a hexahistidine tag into the mammalian expression vector pcDNA3.1 by GenScript.

The plasmid encoding the Spike protein was kindly provided by Dr. Jason S. McLellan (The University of Texas at Austin, USA) (32). Briefly, the trimeric Spike Wuhan-Hu-1 protein (GenBank: MN908947), comprising amino acids 1–1,208, was engineered with proline substitutions at positions 986 and 987, a “GSAS” substitution at the furin cleavage site (residues 682–685), and a C-terminal T4 fibritin trimerization motif. Additionally, the construct included an HRV3C protease cleavage site, a TwinStrepTag, and an 8XHisTag, and it was cloned into the mammalian expression vector pαH.

The plasmids were subsequently transformed by heat shock into TOP10 competent *Escherichia coli* bacteria (Thermo Fisher Scientific). A single colony was then selected and cultured for 16–18 hours in 200 mL of LB medium (Merck) supplemented with 100 μg/mL ampicillin (Sigma-Aldrich). Plasmid DNA extraction was performed using the PureLink™ HiPure Plasmid Maxiprep Kit (Thermo Fisher Scientific) exactly as provided by the manufacturer. The concentrations of the purified plasmids were determined by spectrophotometry (NanoDrop 2000, Thermo Fisher Scientific), and their integrity was analyzed using 0.8% agarose gels.

The mammalian cell-expressed recombinant proteins were transiently expressed in Expi293™ cells (Thermo Fisher Scientific) using the ExpiFectamine™ 293 Transfection Kit according to the manufacturer’s protocol.

After 5 days, the supernatant was collected after centrifugation (3,000 xg for 20 minutes), treated with 1 mM phenylmethylsulfonyl fluoride (PMSF, Thermo Fisher Scientific) and an equal volume of cold 1x phosphate-buffered saline (PBS) was added. The soluble proteins were purified by affinity chromatography using a HisPur™ Ni-NTA column (Thermo Fisher Scientific) and imidazole gradient (5–250 mM) in cold PBS. The eluted fractions were dialyzed with cold 1x PBS and analyzed by 12% SDS-PAGE and a NanoDrop 2000 (Thermo Fisher Scientific) for protein size, purity and concentration.

### Monoclonal antibodies production

C57BL/6 mice (n=3) were intraperitoneally immunized with three doses (on days 0, 14, and 28) of 10 µg of trimeric Spike protein formulated with Poly I:C (50 µg). A booster dose of 10 µg of trimeric Spike protein was given intravenously on day 33. Three days later, splenocytes were harvested and fused with the P3X66Ag8.653 myeloma cell line, which had been pretreated with 8-Azaguanine (Sigma-Aldrich). Fusion was facilitated using polyethylene glycol (Sigma-Aldrich), following standard protocols. Hybridomas were selected in hypoxanthine-aminopterin-thymidine (HAT) medium, and supernatants from hybrid clones were screened for reactivity against RBD of the Spike protein using ELISA. Positive hybridomas were subjected to limiting dilution cloning, expanded, and cryopreserved. Monoclonal antibodies (mAbs) were produced by expanding selected hybridoma clones and collecting their supernatants for further purification using protein G affinity chromatography (GE Healthcare). The mAbs were quantified by spectrophotometry (Nanodrop 2000; Thermo Fisher Scientific), their purity was confirmed by SDS-PAGE analysis, and they were further characterized (unpublished data).

### Immunoblot

Proteins were resolved in 12% SDS-PAGE under reducing conditions and transferred to a nitrocellulose membrane (Hybond-C extra nitrocellulose; GE Healthcare). The membrane was then blocked overnight at 4°C with blocking solution (PBS with Tween 20 (PBST) (0.05% v/v), skim milk (5% w/v) and BSA (2.5% w/v). After each step, the membranes were washed three times for 10 minutes each with PBST. We used in house produced monoclonal antibodies (mAbs) against the RBD (2B9F9, 4H4A2, 4B1D3, and 9G7G8) as primary antibodies, diluted to 1 µg/mL in blocking solution and incubated for 1 hour. HRP-conjugated goat anti-mouse IgG (0.5 µg/mL; KPL) was then added to the blocking solution and incubated for 1 hour, followed by detection using the ECL system (Thermo Fisher Scientific).

For dot blotting, 0.5 µg of each protein was directly added to nitrocellulose membranes (Hybond-C extra nitrocellulose; GE Healthcare), which were subsequently blocked as described. Pooled in-house produced anti-RBD, anti-Spike off-RBD mAb, with specificity to an unknown region outside RBD (1 µg/mL; 2G7D11), and an anti-6x-His tag (0.1 µg/mL; Invitrogen, clone HIS.H8) were used as primary antibodies and incubated in blocking solution for 1 hour. HRP-conjugated goat anti-mouse IgG (0.5 µg/mL; KPL) was then added to the blocking solution and incubated for 1 hour, followed by detection using the ECL system (Thermo Fisher Scientific).

### Mice and immunization

The use of animals in this study was approved by the Institutional Animal Care and Use Committee (IACUC) of UNIFESP (#6264151021), in compliance with the recommendations of Federal Law 11.794 (2008), the Guide for the Care and Use of Laboratory Animals of the Brazilian National Council of Animal Experimentation (CONCEA) and the ARRIVE guidelines (https://arriveguidelines.org).

Seven-week-old female C57BL/6 mice were purchased from CEDEME (Centro de Desenvolvimento de Modelos Experimentais para Medicina e Biologia). CD4 knockout and K18-hACE2 transgenic mice (JAX stock #034860) were purchased from the Institute of Biomedical Sciences (Universidade de São Paulo). All animals were maintained under specific pathogen-free conditions, on a controlled 12:12 light-dark cycle, with *ad libitum* access to water and food.

Mice received two or three doses of vaccine formulations, 15 days apart, via the subcutaneous route at the base of the tail, in a final volume of 100 µL. The Wuhan RBD monomer (10μg or 1μg) and equimolar amounts of Wuhan RBD dimer (2μg) and Delta: Beta:Gamma RBD heterotrimer (3μg) were formulated with adjuvants, including AddaS03 (AS03-like) (1:1v/v), AddaVax (1:1v/v), Poly (I:C) (50 µg/animal), or Alhydrogel 2%, referred to as Alum (1:1 v/v, 500µg aluminum content/dose) + CpG ODN 1826 (50 µg/animal). All adjuvants were obtained from InvivoGen (San Diego, CA, USA). Blood was collected 15 days after each dose, and the serum was separated by centrifugation for further analysis. Fifteen days after the last dose, the mice were euthanized with an overdose of ketamine (300 mg/kg) and xylazine (30 mg/kg) administered intraperitoneally.

### ELISA

High-binding, 96-well plates (Corning, 3590) were coated with 250 ng/well (5 µg/mL) of the RBD monomer, dimer or trimer in PBS overnight at room temperature. Subsequently, the plates were washed with PBST and blocked for 2 hours with 150 µL of PBST containing 1% w/v BSA and 5% w/v skim milk. After washing with PBST, the in-house produced mAbs (2B9F9, 4H4A2, 4B1D3, 9G7G8, and 2G7D11) or serum from immunized mice were serially diluted and incubated for 2 hours. Following another round of washing, 50 µL of HRP-conjugated goat anti-mouse total IgG (0.1 µg/mL; KPL) or HRP-conjugated goat anti-mouse IgG1, IgG2b or IgG2c (0.25 μg/mL; Southern Biotech) was added. After 2-hour incubation, the enzymatic reaction was initiated by adding 1 mg/mL o-phenylenediamine (Sigma-Aldrich) diluted in phosphate–citrate buffer (pH 5) containing 0.03% v/v hydrogen peroxide (Sigma-Aldrich). The reaction was stopped by adding a solution of 4 N H_2_SO_4_. Finally, the plates were read using an ELISA reader (EnSpire Multimode Plate Reader; PerkinElmer) at 492 nm (OD492). The antibody titer was determined as the highest dilution of serum that presented an OD_492nm_ between 0.1 and 0.2.

### Antibody affinity assay

ELISA plates were coated with 250 ng/well (5 µg/mL) of the RBD monomer, dimer or trimer in PBS overnight at room temperature. The pooled serum from mice was diluted to achieve an OD_492nm_ between 0.9 and 1.0 and plated in triplicate. After a 2-hour incubation, the chaotropic agent ammonium thiocyanate (Sigma-Aldrich) was added to each row at serially diluted concentrations ranging from 8 M to 0.125 M, and the plates were incubated for 15 minutes. The percentage of affinity was determined as OD_492nm_ in the presence of ammonium thiocyanate x 100)/OD_492nm_ in the presence of PBS.

### Pseudovirus production and pseudovirus neutralization assay

Pseudoviruses expressing the SARS-CoV-2 Spike protein from the Wuhan Hu-1 strain and Omicron BA.2 were generated following a previously described method (33). Briefly, HEK293T cells were transfected with plasmids encoding firefly luciferase (pNL43 ΔEnv-NanoLuc) and either the SARS-CoV-2 Wuhan S protein (pSARS-CoV-2-Swuhan_trunc_) or the Omicron BA.2 S protein (pSARS-CoV-2-Somicron_trunc_), kindly provided by Dr. Paul D. Bieniasz (The Rockefeller University). After 48 hours, the supernatants were collected, centrifuged, and stored at -80°C. The luciferase activity of each production was assessed by relative light units (RLU) and the pseudoviral titers were defined.

For the neutralization assay, HT1080-hACE2 cells (kindly provided by Dr. Paul D. Bieniasz, The Rockefeller University) were seeded at a density of 1x10^4^ cells per well in DMEM supplemented with 5% FBS, 1 mM sodium pyruvate, 2 mM L-glutamine, and 5 µg/mL blasticidin (all from Gibco). The cells were then incubated at 37°C with 8% CO_2_ for 24 hours in a 96-well plate (Corning, 3809). Heat-inactivated sera (56°C for 30 minutes) from immunized mice were mixed at a 1:1 ratio with pseudoviruses at a dilution of 10^7^ RLU, and the mixture was incubated for 1 hour at 37°C. After the incubation period, the serum-virus mixture was added to HT1080-hACE2 cells that had been previously seeded and incubated at 37°C for 48 hours. The luminescence emission was measured using Luciferase Assay System kits (Promega). The neutralizing titer (NT50) was defined as the serum dilution that produced a 50% reduction in relative light units (RLU) compared to the virus control wells, thereby reflecting the neutralizing capacity of each serum sample.

### Virus neutralization test

The neutralizing antibody (nAb) titers against SARS-CoV-2 were quantified following the method described previously (34). Vero cells (ATCC CCL-81) were seeded at a density of 5x10^4^ cells per well in 96-well culture plates. After 24 h, the cells were exposed to 1x10^3^ TCID_50_/mL SARS-CoV-2 strains, which included Wuhan (GISAID: EPI_ISL_2499748), Delta (GISAID EPI_ISL_2965577), Gamma (GISAID EPI_ISL_1060981) and Omicron BA.1 (GISAID: EPI_ISL_6794907), which had been previously incubated with 2-fold diluted heat-inactivated immunized mouse serum samples ranging from 1:20 to 1:1,280. After a 3-day incubation period, all the wells were examined under optical microscopy for characteristic SARS-CoV-2 cytopathic effects (CPEs). The absence of cytopathic effects, particularly in the 1:20 dilution sample, was considered indicative of the presence of neutralizing antibodies against SARS-CoV-2. The VNT_100_ was defined as the highest dilution of serum that completely (100%) inhibits cytopathic effects in cell culture. All procedures were conducted in a biosafety level 3 laboratory (BSL-3), in accordance with WHO recommendations.

### Viral challenge

For viral challenge, K18-hACE2 transgenic mice were immunized with a 2-dose vaccination regimen. After seroconversion by ELISA, the animals were transferred to a BSL-3 animal facility. Subsequently, mice were intranasally challenged with 2.8x10^5^ TCID_50_/mL of the SARS-CoV-2 Wuhan strain in a final volume of 30 µL under isoflurane anesthesia. The groups were monitored daily for signs and symptoms. The humane endpoint was defined as a weight loss higher than 25%, drastic reduction of mobility or significant reduction of the response to stimuli or clinically significant respiratory distress. Animals meeting these criteria were humanely euthanized under anesthesia overdose. At six days post infection (dpi), all the mice were euthanized, and lung and brain tissues were collected for further analysis.

### Molecular detection of viral load by RT-qPCR

The viral load was quantified in the brain and lung tissues. Initially, the organs were collected, weighed, and placed in tubes containing 500 μL of DMEM supplemented with 1% penicillin-streptomycin (all from Gibco) and 1 mm glass beads. The tubes were then homogenized twice using a Qiagen TissueLyser II at 30 Hz for 2 minutes, followed by centrifugation (2,000 xg for 30 seconds). Total RNA was extracted using the MagMAX™ Viral/Pathogen II (MVP II) Nucleic Acid Isolation Kit (Thermo Fisher Scientific). The presence of SARS-CoV-2 RNA was detected by amplifying the SARS-CoV-2 E gene using the AgPath-ID™ One-Step RT-PCR Reagent (Thermo Fisher Scientific) with the following probe and primers: Probe: FAM-ACACTAGCCATCCTTACTGCGCTTCG-BQ; Primers: F 5’ ACAGGTACGTTAATAGTTAATAGCGT 3’ and R 5’ ATATTGCAGCAGTAC-GCACACA 3’. Brain and lung samples were processed in duplicate, and the results are expressed as TCID_50_/mL/g of tissue.

### Splenocyte isolation

Fifteen days after the last dose, the mice were euthanized, and the spleens were aseptically removed and processed in RPMI medium (Gibco) using a cell strainer (100μm, Falcon). The cell suspension was subjected to red blood cell lysis with ammonium- chloride- potassium (ACK) for 2 minutes. The reaction was stopped by adding R10 medium (RPMI 1640 supplemented with 10% fetal bovine serum (FBS), 1% v/v L-glutamine, 1% v/v sodium pyruvate, 1% v/v non-essential amino acids, 1% v/v penicillin-streptomycin, and 5 × 10–^5^ M 2-mercaptoethanol (all from Gibco). Subsequently, the cells were washed, resuspended in R10 medium, and counted using 0.2% Trypan blue solution in a Neubauer counting chamber for concentration determination and viability assessment.

### Cell sorting

For cell sorting, we collected splenocytes from mice immunized with three doses of the RBD dimer together with AddaS03 + Poly I:C and from control mice that received only the adjuvants. Splenocytes were stained with anti-CD3 PE (BioLegend, cat. 100308; clone 145-2C11), anti-CD4 BV421 (BioLegend, cat. 100563; clone RM4-5) and anti-CD8 PE Cy7 (BioLegend, cat. 100722; clone53-6.7) for 30 min at 4°C in 1 mL of MACS buffer (0.5% BSA, 2 mM of EDTA in PBS1x). After sorting on a FACS Aria II (BD Biosciences), we achieved a purity greater than 97% for all samples. Sorted CD4^+^ (CD3^+^ CD4^+^CD8^-^) and CD8^+^ (CD3^+^CD4^-^CD8^+^) T cells were used in ELISpot.

### Peptide library

A library comprising 30 peptides of 17 amino acids in length, with 10 overlapping amino acids, covering the entire RBD sequence from the USA-WA1/2020 strain was synthetized (**Table 1**; BEI Resources, cat. NR-52402). The peptides were arranged into 12 pools using a matrix strategy generated by the DeconvoluteThis! Software (v. 2.0) (35), resuspended in dimethyl sulfoxide (DMSO) and stored at −20°C until use. Each pool consisted of five peptides. In pools 1 to 6, the peptides were arranged in ascending order. In pools 7 to 12, the same peptides were randomly allocated, ensuring that each peptide was present in two distinct pools (**Table 2**).

**Table 1:** Peptide library.

Peptide number	Sequence and Spike position
Pep 1	316-SNFRVQPTESIVRFPNI-332
Pep 2	323-TESIVRFPNITNLCPFG-339
Pep 3	330-PNITNLCPFGEVFNATR-346
Pep 4	337-PFGEVFNATRFASVYAW-353
Pep 5	344-ATRFASVYAWNRKRISN-360
Pep 6	351-YAWNRKRISNCVADYSV-367
Pep 7	358-ISNCVADYSVLYNSASF-374
Pep 8	365-YSVLYNSASFSTFKCYG-381
Pep 9	372-ASFSTFKCYGVSPTKLN-388
Pep 10	379-CYGVSPTKLNDLCFTNV-395
Pep 11	386-KLNDLCFTNVYADSFVI-402
Pep 12	393-TNVYADSFVIRGDEVRQ-409
Pep 13	400-FVIRGDEVRQIAPGQTG-416
Pep 14	407-VRQIAPGQTGKIADYNY-423
Pep 15	414-QTGKIADYNYKLPDDFT-430
Pep 16	421-YNYKLPDDFTGCVIAWN-437
Pep 17	428-DFTGCVIAWNSNNLDSK-444
Pep 18	435-AWNSNNLDSKVGGNYNY-451
Pep 19	442-DSKVGGNYNYLYRLFRK-458
Pep 20	449-YNYLYRLFRKSNLKPFE-465
Pep 21	456-FRKSNLKPFERDISTEI-472
Pep 22	463-PFERDISTEIYQAGSTP-479
Pep 23	470-TEIYQAGSTPCNGVEGF-486
Pep 24	477-STPCNGVEGFNCYFPLQ-493
Pep 25	484-EGFNCYFPLQSYGFQPT-500
Pep 26	491-PLQSYGFQPTNGVGYQP-507
Pep 27	498-QPTNGVGYQPYRVVVLS-514
Pep 28	505-YQPYRVVVLSFELLHAP-521
Pep 29	512-VLSFELLHAPATVCGPK-528
Pep 30	519-HAPATVCGPKKSTNLVK-535

Peptides covering the entire RBD protein sequence. The first and last numbers flanking each sequence represent the peptide position in the Spike protein.

**Table 2:** Peptide pools.

Pools Matrix			
Pool 1	Pep 1	Pool 7	Pep 1
	Pep 2		Pep 6
	Pep 3		Pep 11
	Pep 4		Pep 16
	Pep 5		Pep 21
Pool 2	Pep 6	Pool 8	Pep 2
	Pep 7		Pep 7
	Pep 8		Pep 12
	Pep 9		Pep 22
	Pep 10		Pep 26
Pool 3	Pep 11	Pool 9	Pep 3
	Pep 12		Pep 8
	Pep 13		Pep 13
	Pep 14		Pep 17
	Pep 15		Pep 27
Pool 4	Pep 16	Pool 10	Pep 4
	Pep 17		Pep 9
	Pep 18		Pep 14
	Pep 19		Pep 18
	Pep 20		Pep 28
Pool 5	Pep 21	Pool 11	Pep 5
	Pep 22		Pep 10
	Pep 23		Pep 15
	Pep 24		Pep 19
	Pep 25		Pep 29
Pool 6	Pep 26	Pool 12	Pep 20
	Pep 27		Pep 23
	Pep 28		Pep 24
	Pep 29		Pep 25
	Pep 30		Pep 30

Peptides were arranged in 12 pools using the software DeconvoluteThis! Software (v. 2.0).

### T cell ELISpot assay

To detect specific IFNγ producing cells, we used the ELISpot Mouse IFNγ set (BD Biosciences). Flat-bottom, 96-well PVDF plates (Millipore, MAIPS4510) were treated with 25 µL of 35% ethanol for 30 seconds and washed three times with PBS under sterile conditions. The plates were subsequently coated overnight at 4°C with purified anti-IFNγ in PBS, following the manufacturer’s instructions (BD Biosciences, cat. 51-2525KZ). On the following day, the plates were washed and blocked with R10 medium for 2 hours at room temperature. Splenocytes were plated at 5x10^5^ cells/well in R10 medium supplemented with 30 U/mL recombinant IL-2 (ZODIAC) and stimulated with peptide pools (10 μg/mL), individual peptides (10 μg/mL), RBD protein (5 μg/mL), or Concanavalin A (ConA, Sigma-Aldrich) as a positive control (2.5 μg/mL). For ELISpots with sorted cells, purified CD4^+^ and CD8^+^ T cells were plated at 1x10^5^ cells/well together with 3x10^5^ total splenocytes/well from naive mice, in R10 medium with IL-2 (30 U/mL) and stimulated as described above. The cells were then incubated for 18 hours at 37°C with 5% CO_2_. After washing with PBS, biotin-conjugated anti-mouse IFNγ was added to PBS containing 10% FBS and incubated for 2 hours. The plates were washed, and streptavidin-HRP in PBS containing 10% FBS was added, followed by 1 hour incubation. The reaction was developed using 3-amino-9-ethylcarbazole (AEC, BD Biosciences) for 20–40 minutes in the dark. Subsequently, the plates were washed with tap water and left to dry overnight. Spots were counted using the AID Classic ELISPOT Reader (Autoimmun Diagnostika GmbH). The spots from unstimulated wells were subtracted and the number of spot forming units (SFUs) per 10^6^ splenocytes was calculated.

### B cell ELISpot assay

Flat-bottom, 96-well PVDF plates (Millipore, MAIPS4510) were treated with 35% ethanol and coated under sterile conditions with 500 ng/well of RBD dimer (5 µg/mL) in PBS overnight at room temperature. The following day, the bone marrow cells were first treated with ACK buffer to lyse red blood cells, platted (5x10^5^/well) in 200 µL of R10 medium and incubated for 12 hours at 37°C and 5% of CO_2_. The plates were washed with PBS and HRP-conjugated goat anti-mouse IgG (0.1 µg/mL; KPL), IgG1 (2.5 μg/mL; Southern Biotech) or IgG2c (2.5 μg/mL; Southern Biotech) was added. After 2 hours, the plates were washed, and the reaction was developed with AEC (BD Biosciences).

### Statistical analysis

The statistical significance (p value) was determined by one-way or two-way ANOVA followed by Tukey’s *post hoc* test for multiple comparisons. For VNTs, p values were calculated with Kruskal-Wallis followed by Dunn’s *post hoc* test for multiple comparisons. To analyze the survival rate, Log-rank test was used. All the statistical analyses and graphics were generated using GraphPad Prism version 10.0 software. The details can be found in the figure legends.

## Results

### Protein characterization

The secreted recombinant RBDs were assessed by gel electrophoresis for size and purity and exhibited patterns consistent with those of the expected constructs: the monomer presented ~40 kDa, the dimer ~80 kDa, and the trimer ~120 kDa (**Supplementary Figure 1A**). Western blot analysis with mAbs against the RBD (2B9F9, 4H4A2, 4B1D3, and 9G7G8) produced in-house demonstrated that all three RBD constructs, and the Spike protein were recognized as a single band (**Supplementary Figure 1B**). Dot blots were performed to evaluate proteins in their native conformation using anti-RBD and anti-Spike off-RBD (2G7D11) mAbs, and an anti-6x-His tag. The RBD-directed mAbs (2B9F9, 4H4A2, 4B1D3, and 9G7G8) and the anti-His tag antibody recognized all recombinant RBDs and the Spike protein (**Supplementary Figure 1C**). As expected, the 2G7D11 mAb, an antibody specific to an unknown region outside RBD, only recognized the Spike protein. Furthermore, ELISAs with the mAbs were performed, and the resulting curves for each protein presented a dose-response pattern (**Supplementary Figures 1D-F**). These findings confirmed the integrity of the recombinant proteins.

### AddaS03 is the most effective adjuvant for inducing neutralizing antibodies

**Figure 1:**
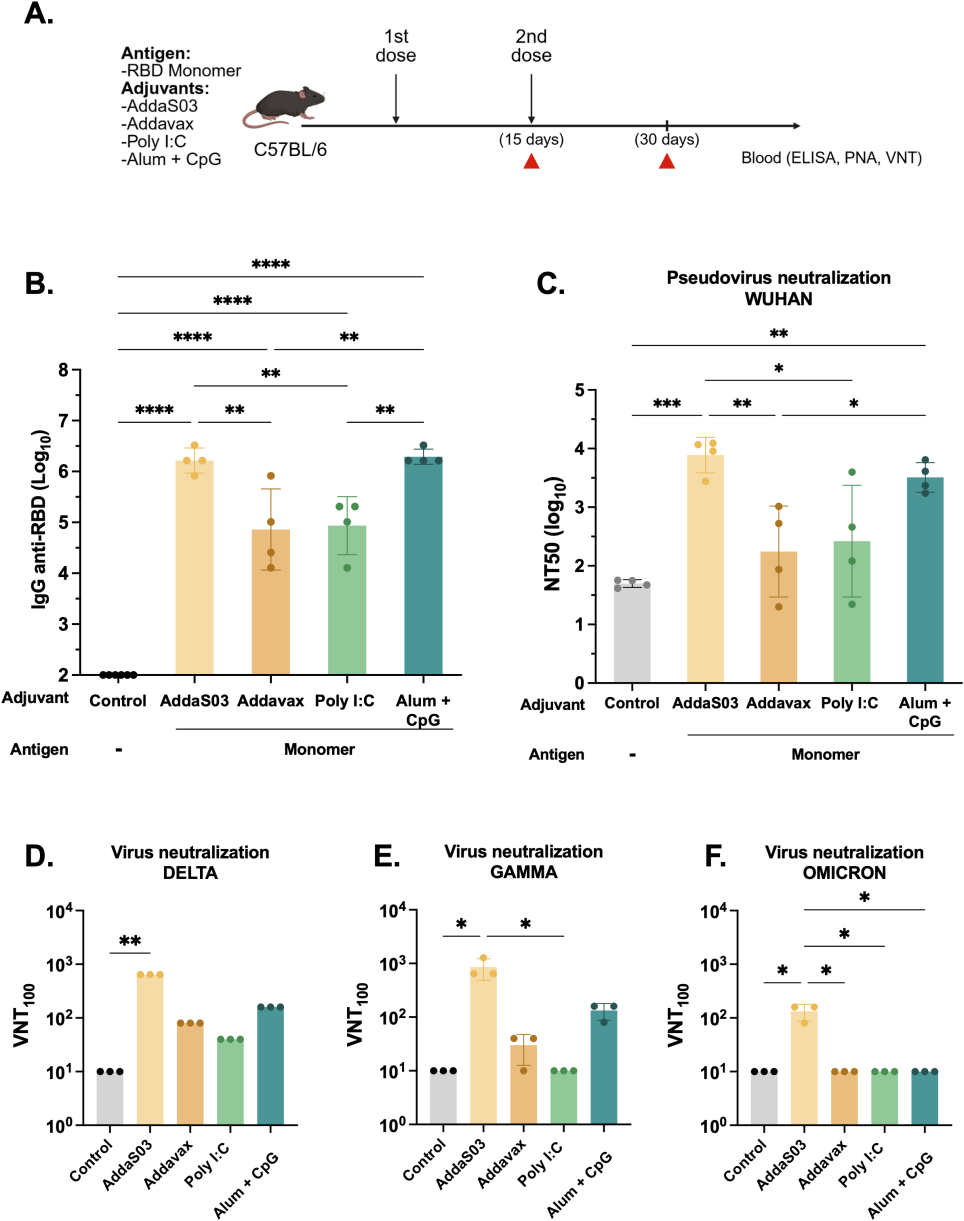
AddaS03 is the most potent adjuvant to induce humoral response. **(A)** Study design. C57BL/6 mice (n=4) were immunized subcutaneously with two doses of the RBD monomer together with AddaS03, AddaVax, Poly I:C or Alum + CpG 15 days apart. The control groups received only adjuvants (Alum+ CpG, AddaS03, Poly I:C). **(B)** Anti-RBD total IgG titers 15 days after two doses. Data represent the mean ± SD of antibody titers in log_10_ scale. **(C)** NT50 pseudovirus neutralization assay (PNA) against Wuhan pseudovirus after two doses. Serum neutralizing antibody responses after two doses as measured by live virus neutralization assay (VNT_100_) against **(D)** Delta, **(E)** Gamma and **(F)** Omicron BA.1 variants of SARS-CoV-2. For ELISA and PNA, serum of each animal was assayed individually. For the VNT_100_, serum of each group was pooled and tested in triplicate. Data represent the mean ± SD. b, c: Statistical analysis was determined by one-way ANOVA followed by Tukey *post-hoc* test.; d, e, f: Statistical analysis was determined by Kruskal-Wallis followed by Dunn’s *post hoc* test. *p < 0.05, **p < 0.01, ***p < 0.001, and ****p < 0.0001. Only statistically significant comparisons are depicted.

To evaluate the influence of different adjuvant formulations, we immunized C57BL/6 mice with a two-dose regimen with the RBD monomer (10 µg) formulated with AddaS03, AddaVax, Poly I:C, or Alhydrogel 2%, referred to as Alum, + CpG. PBS plus adjuvants were given as controls. Serum samples were collected 15 days after the last immunization as indicated in **Figure 1A**. Mice immunized with AddaS03 and Alum + CpG presented significantly higher RBD-specific IgG titers than those immunized with AddaVax or Poly I:C (mean of AddaS03 vs AddaVax and Poly I:C, p < 0.01; mean of Alum + CpG vs AddaVax and Poly I: C, p < 0.01) (**Figure 1B**). Subsequently, we assessed neutralizing antibody (nAb) titers using a pseudovirus neutralization assay (PNA) and live virus neutralization test (VNT_100_). AddaS03 induced superior neutralizing activity against Wuhan pseudovirus (**Figure 1C**) and against Gamma, Delta, and Omicron BA.1 VOCs in the VNT_100_ (**Figures 1D–F**. Based on these results, AddaS03 was selected for subsequent experiments.

### RBD dimer and trimer elicit high nAb titers

**Figure 2:**
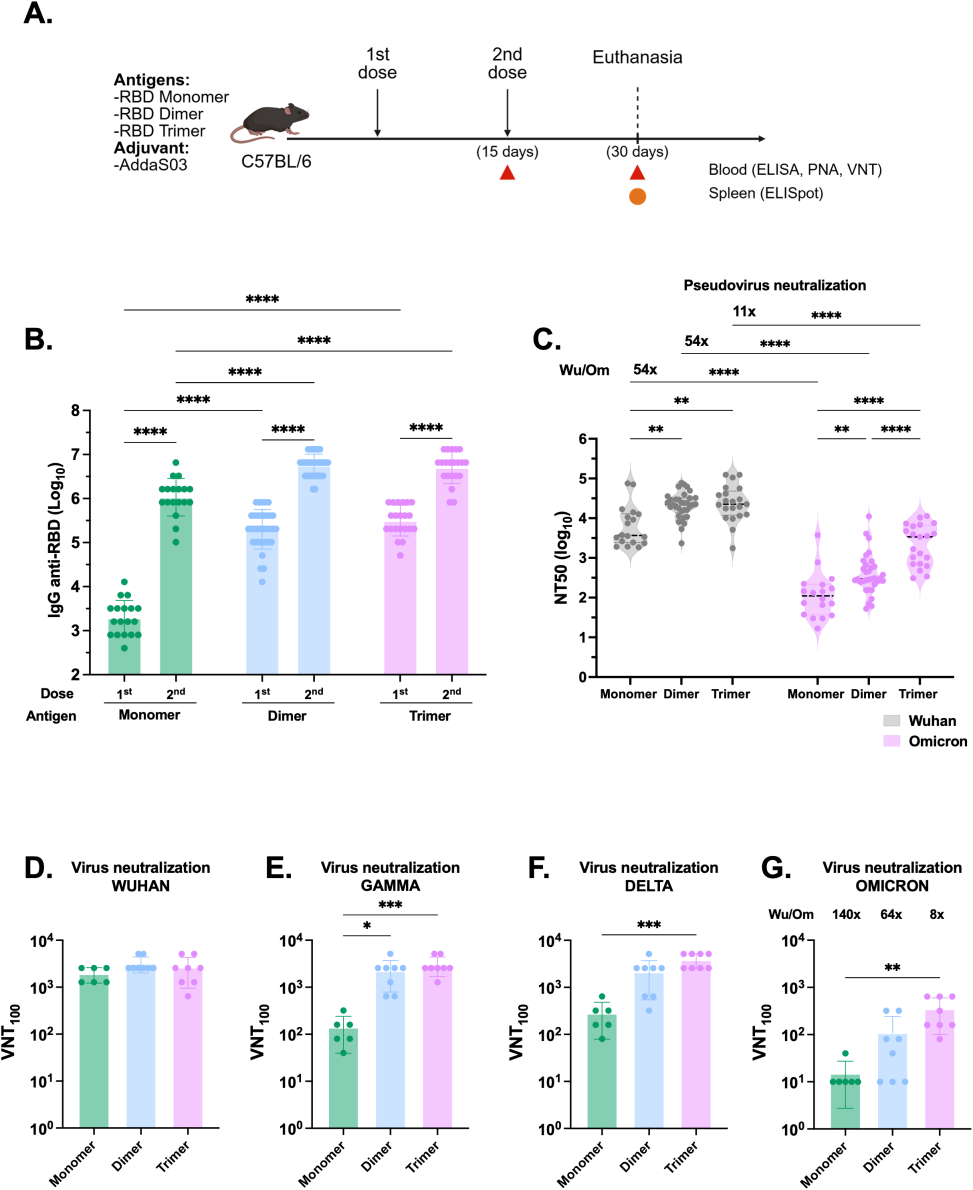
The RBD dimer and trimer are superior immunogens to induce humoral responses. **(A)** Study design. C57BL/6 mice were immunized subcutaneously with two doses of RBD monomer (n=18), dimer (n=34) or trimer (n=21) combined with AddaS03. **(B)** Anti-RBD total IgG titers 15 days after one and two doses. Data represent the mean ± SEM. **(C)** NT50 PNA against Wuhan and Omicron BA.2–15 days after two doses. Data represent the median ± interquartile range. **(D)** VNT_100_ against Wuhan, **(E)** Gamma, **(F)** Delta and **(G)** Omicron BA.1–15 days after two doses. For ELISA and PNA, serum of each animal was assayed individually. Data are from 7 independent experiments. For VNT, the samples from monomer RBD were derived from two independent experiments and assayed in triplicate (n=6). For dimer and trimer RBDs, samples came from three independent experiments—two performed in triplicate and one in duplicate (n=8 each). Data represent the mean ± SEM. b, c: Statistical analysis was determined by two-way ANOVA followed by Tukey *post-hoc* test.; d, e, f, g: Statistical analysis was determined by Kruskal-Wallis followed by Dunn’s *post hoc* test. *p < 0.05, **p < 0.01, ***p < 0.001, and ****p < 0.0001. Wu/Om = Wuhan/Omicron fold of decay. Only statistically significant comparisons are depicted.

To further compare the immunogenicity of the RBD proteins, we immunized C57BL/6 mice with a low two-dose regimen of monomer (1µg) and equimolar amounts of dimer and trimer in the presence of AddaS03 (**Figure 2A**). Fifteen days post boost, mice immunized with dimer and trimer exhibited significantly higher antibody titers compared to monomer (p < 0.0001), with no significant difference between the dimer and trimer (**Figure 2B**). Moreover, both dimer and trimer markedly enhanced the neutralization capacity compared to monomer in the Wuhan PNA (p < 0.01). Notably, the trimer induced higher nAb titers against Omicron BA.2 pseudovirus (p < 0.0001) (**Figure 2C**). The fold decay of Wuhan compared to Omicron PNA was calculated, with the trimer displaying an 11-fold decay, while both the monomer and dimer presented a 54-fold decay.

In VNT_100_, no difference was observed between proteins against the Wuhan strain (**Figure 2D**), whereas both the dimer and trimer displayed superior neutralization capacity against the Gamma variant (**Figure 2E**). Additionally, the trimer performed better against Delta and Omicron BA.1 VOCs (**Figures 2F**, **G**). The fold decay of Wuhan compared to Omicron in VNT_100_ was also calculated and, similarly to PNA, was lowest in the trimer (8-fold) while the monomer and dimer exhibited a higher drop in the titers against the VOCs (140- and 64-fold decay, respectively). Overall, these findings demonstrated that immunization with dimer and trimer elicited higher and broader humoral responses.

To assess the T cell response, we euthanized the animals 15 days after the second dose and harvested the splenocytes to detect specific IFNγ producing cells by ELISpot. Cells were stimulated with pools of overlapping peptides comprising the full length RBD organized in a matrix and the respective proteins. However, after 18 hours of incubation, the stimuli induced negligible numbers of IFNγ producing T cells, with a magnitude lower than 20 SFU/10^6^ splenocytes (**Supplementary Figure 2**).

### The immune response induced by AddaS03-adjuvanted RBD immunization depends on CD4 T cells

To evaluate whether RBD humoral responses were T cell dependent, we immunized wild-type (WT) C57BL/6 and CD4 knockout (KO) mice with monomeric RBD as a model antigen in combination with AddaS03, as outlined in **Supplementary Figure 3A**. As expected, WT mice exhibited specific IgG, whereas CD4 KO mice presented no detectable titers, similar to control group that received only AddaS03 (**Supplementary Figure 3B**). These findings strongly suggest that the immune response to RBD immunization is dependent on CD4 T cells.

### Immunization with RBD homodimer and heterotrimer protects against viral replication in the lungs after challenge

**Figure 3:**
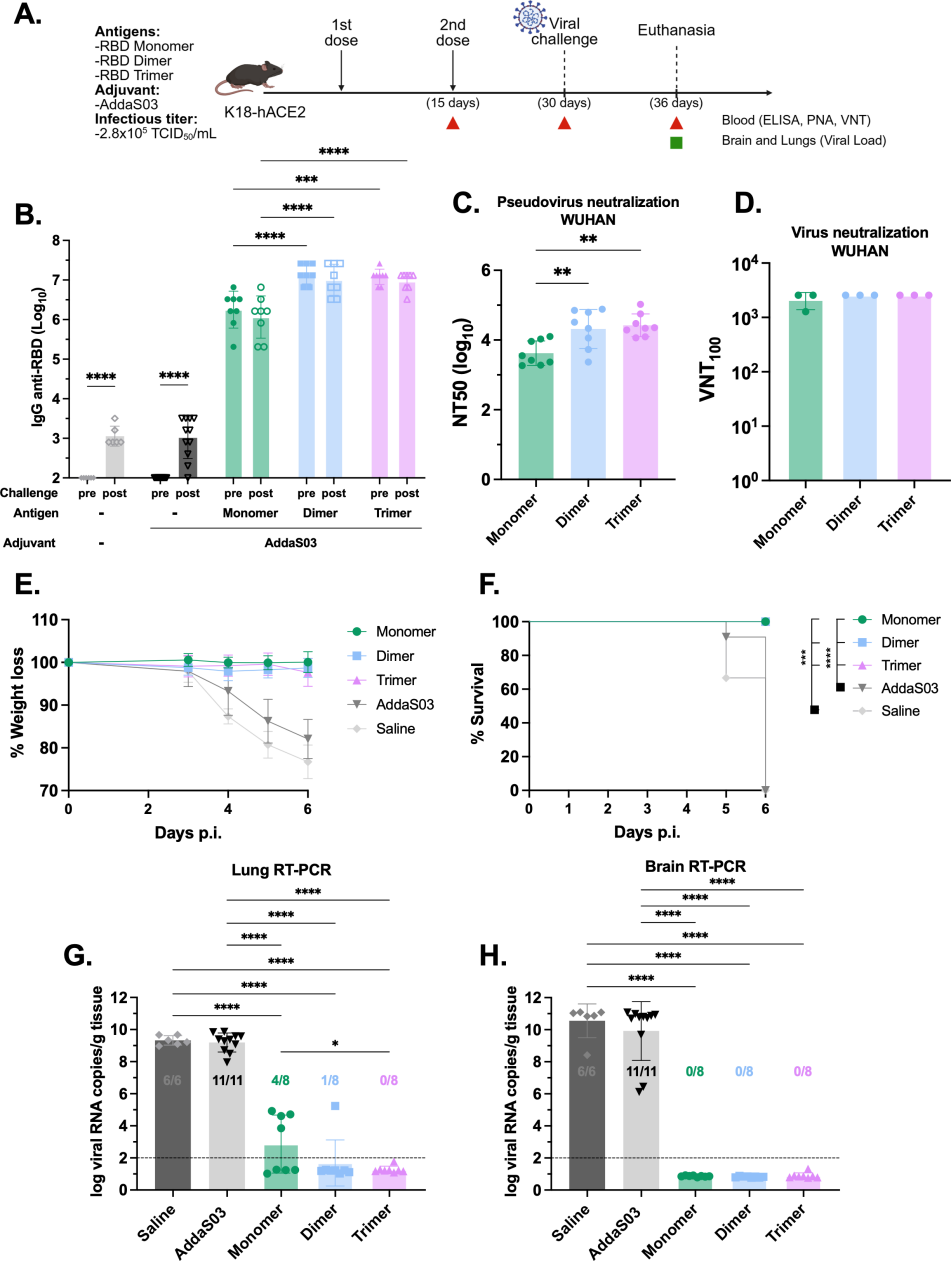
Lethal SARS-CoV-2 challenge in K18-hACE2 mice following immunization with RBD monomer, dimer and trimer in the presence of AddaS03. **(A)** Study design. K18-hACE2 mice were immunized subcutaneously with two doses of RBD monomer (n=8), dimer (n=8) or trimer (n=8) together with AddaS03. Control groups received AddaS03 only (n=11) or saline (n=6). Fifteen days after the second dose, mice were intranasally challenged with SARS-CoV-2 (2.8x10^5^ TCID_50_/mL) and accompanied for 6 days followed by euthanasia. **(B)** Total anti-RBD IgG titers 15 days after two doses. Full circles represent pre-challenge titers while empty circles describe post-challenge titers. Data represent the mean ± SD. **(C)** NT50 PNA against Wuhan 15 days after two doses. Data represent the median ± interquartile range. **(D)** VNT_100–_15 days after two doses against Wuhan. For ELISA, PNA and qPCR, samples from each animal were assayed individually. For VNT, serum of each group was pooled and used in triplicate. Data represent the mean ± SD. **(E)** Weight loss. **(F)** Survival rate. **(G)** Lung and **(H)** Brain viral load after challenge. Dashed line represents the threshold of detection. Data represent the mean ± SD. **(B)** Statistical analysis was determined by two-way and **(C, G, H)** by one-way ANOVA followed by Tukey post-hoc test; **(D)** Statistical analysis was determined by Kruskal-Wallis followed by Dunn’s post hoc test; **(F)** Statistical analysis was determined by Log-Rank test. *p < 0.05, **p < 0.01, ***p < 0.001, and ****p < 0.0001. Only statistically significant comparisons are depicted.

To further explore the protective capacity of the recombinant RBDs *in vivo*, we conducted a lethal SARS-CoV-2 challenge. K18-hACE2 transgenic mice received a two-dose regimen of 1 µg monomer and equimolar amounts of dimer and trimer in the presence of AddaS03 (**Figure 3A**). AddaS03 alone or saline were used as controls. Fifteen days after the second dose, a similar pattern of humoral response was observed, with dimer and trimer eliciting higher IgG titers and nAbs titers (**Figures 3B-D**). Subsequently, mice were intranasally challenged with Wuhan strain and monitored for 6 days. By day 4, animals in the AddaS03 and saline groups began to exhibit progressive weight loss and clinical signs, and by day 5 one animal in the AddaS03 group succumbed to infection, while two animals in the saline group also died (**Figures 3E, F**). Although still alive at day 6, the remaining animals in the AddaS03 and saline groups met the endpoint criteria and were humanely euthanized. In contrast, mice immunized with all three proteins displayed no significant weight loss and clinical signs and survived throughout the experiment. At 6 days post-infection (d.p.i), all surviving animals were euthanized, and lung and brain tissue were harvested for virus detection. Quantification of SARS-CoV-2 viral load in the lung and brain showed that all mice from the AddaS03 and saline groups exhibited high RNA levels in both tissues (**Figures 3G**, **H**). Notably, the monomer group exhibited a significantly higher viral load in the lungs compared to trimer-immunized mice (p < 0.05) (**Figure 3G**). However, while 100% and 87.5% of the mice in the trimer and dimer-immunized groups, respectively, were protected from the presence of viral RNA in the lungs, only 50% (4/8) of the mice in the monomer group were protected (4 mice displayed detectable viral load). Immunization with all three proteins provided protection from viral replication in the brain (**Figure 3H**).

Taken together, the findings suggest that all three RBD formulations in the presence of AddaS03 confer protection, but the RBD dimer and trimer are superior protective immunogens against lethal SARS-CoV-2 infection.

### The combination of AddaS03 + Poly I:C performs similar to AddaS03 and superior to Poly I:C in humoral assays

**Figure 4:**
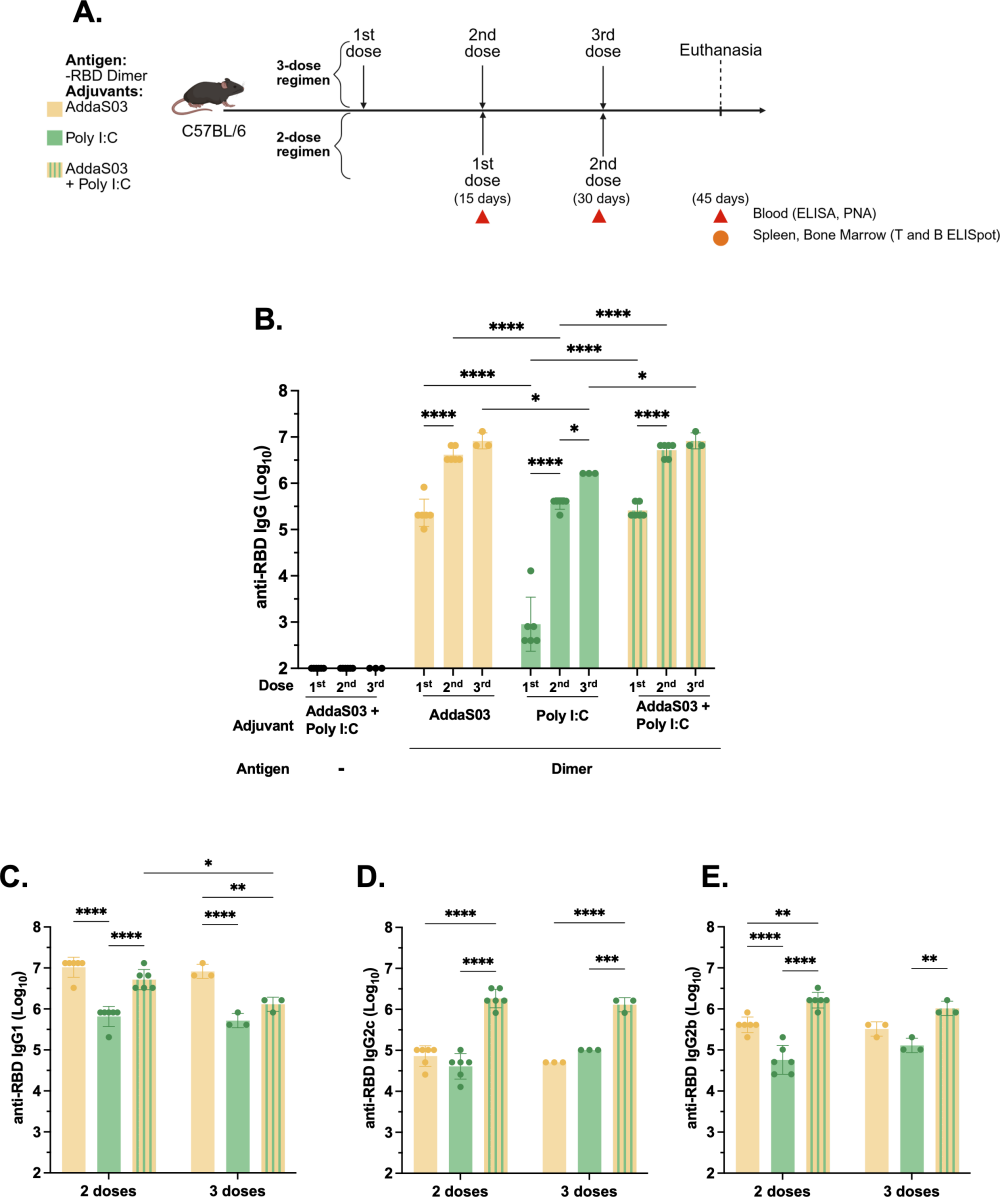
Humoral analysis after two or three doses of RBD dimer with AddaS03, Poly I:C or AddaS03 + Poly I:C mixture. **(A)** Study design. C57BL/6 mice (n=3) were immunized subcutaneously with two or three doses of RBD dimer together with AddaS03, Poly I:C or AddaS03 + Poly I:C mixture. Control group received AddaS03 + Poly I:C only. **(B)** Total anti-RBD IgG titers 15 days after each dose. **(C)** Anti-RBD IgG1 **(D)** IgG2c and **(E)** IgG2b subtypes after two or three doses. For ELISA and PNA, the serum of each animal was assayed individually. Data represent the mean ± SD. Statistical analysis was determined by two-way ANOVA followed by Tukey *post-hoc* test. *p < 0.05, **p < 0.01, ***p < 0.001, and ****p <0.0001. Only statistically significant comparisons are depicted.

The use of AddaS03 alone as an adjuvant failed to induce IFNγ producing T cells, which are highly beneficial against intracellular pathogens such as SARS-CoV-2. To overcome this challenge, we explored the possible additive effect of combining the adjuvant AddaS03 with Poly I:C, while using RBD dimer as a model antigen. Concurrently, we investigated the effect of an additional dose (**Figure 4A**).

After two doses, AddaS03 and the combination of AddaS03+ Poly I:C induced the highest IgG titers (**Figure 4B**). However, there was no significant difference when a third dose was administered in the presence of these adjuvants (**Figure 4B**). In contrast, there was a slight increase in antibody titers after a third dose in the group that received the antigen with Poly I:C alone. We also analyzed IgG subtypes and, similar to total IgG levels, two doses of RBD dimer with AddaS03 and AddaS03 + Poly I:C induced superior titers of IgG1 (**Figure 4C**). Regarding IgG2c (**Figure 4D**), the combination of AddaS03 + Poly I:C generated significantly higher titers (p = <0.0001) than the adjuvants alone, with no distinction between two or three doses, a pattern also observed for IgG2b (**Figure 4E**).

**Figure 5:**
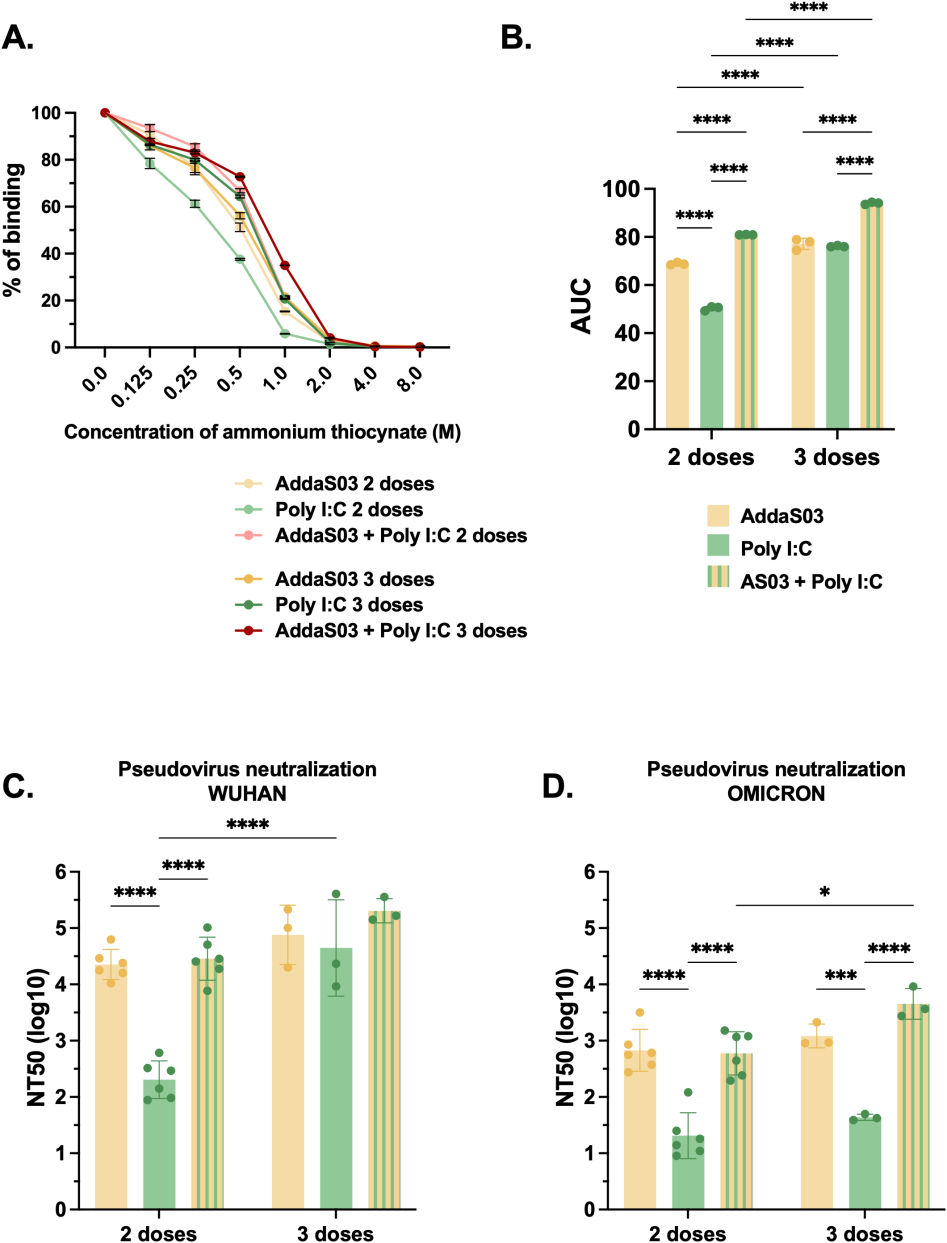
Affinity and neutralization after two or three doses of RBD dimer together with AddaS03, Poly I:C or AddaS03 + Poly I:C mixture. C57BL/6 mice were immunized subcutaneously with two or three doses of RBD dimer together with AddaS03, Poly I:C or AddaS03 + Poly I:C mixture. **(A)** Antibody affinity of pooled mouse sera after incubation with increasing concentrations of ammonium thiocyanate. **(B)** Area under the curve of the affinity assay. **(C)** NT50 PNA against Wuhan and **(D)** Omicron BA.2–15 days after two or three doses. For PNA, serum of each animal was assayed individually. For affinity ELISA, serum of each group was pooled and used in triplicate. Data represent the mean ± SD. Statistical analysis was determined by two-way ANOVA followed by Tukey *post-hoc* test. *p < 0.05, ***p < 0.001, and ****p < 0.0001. Only statistically significant comparisons are depicted.

To assess potential differences in antibody affinity, we used ammonium thiocyanate as a chaotropic agent. After two doses, antibodies from the group immunized with RBD dimer together with AddaS03 + Poly I:C exhibited higher affinity to the antigen (p < 0.0001) (**Figures 5A**, **B**). After a third dose, the difference was more prominent (p<0.0001).

To investigate neutralization ability, we conducted a PNA for both Wuhan and Omicron BA.2 pseudoviruses. The mixture AddaS03 + Poly I:C performed similarly to AddaS03, with both being superior to Poly I:C alone. There was no significant difference between two and three doses in Wuhan PNA, except for the Poly I:C group (**Figure 5C**). For Omicron PNA, AddaS03 and AddaS03 + Poly I:C mixture also performed similarly, displaying superior neutralization titers. However, while AddaS03 did not increase after the third dose, AddaS03 + Poly I:C displayed a slightly superior neutralizing capacity (**Figure 5D**).

These data support the notion that the combination of AddaS03 with Poly I:C generated humoral response similar to that of AddaS03 alone, with high antibody titers and neutralization activity. AddaS03+PolyI:C induced superior antibody affinity and a higher IgG2c response compared to the individual adjuvants.

### Mixture of AddaS03 and Poly I:C induces superior memory plasma cell responses in bone marrow

**Figure 6:**
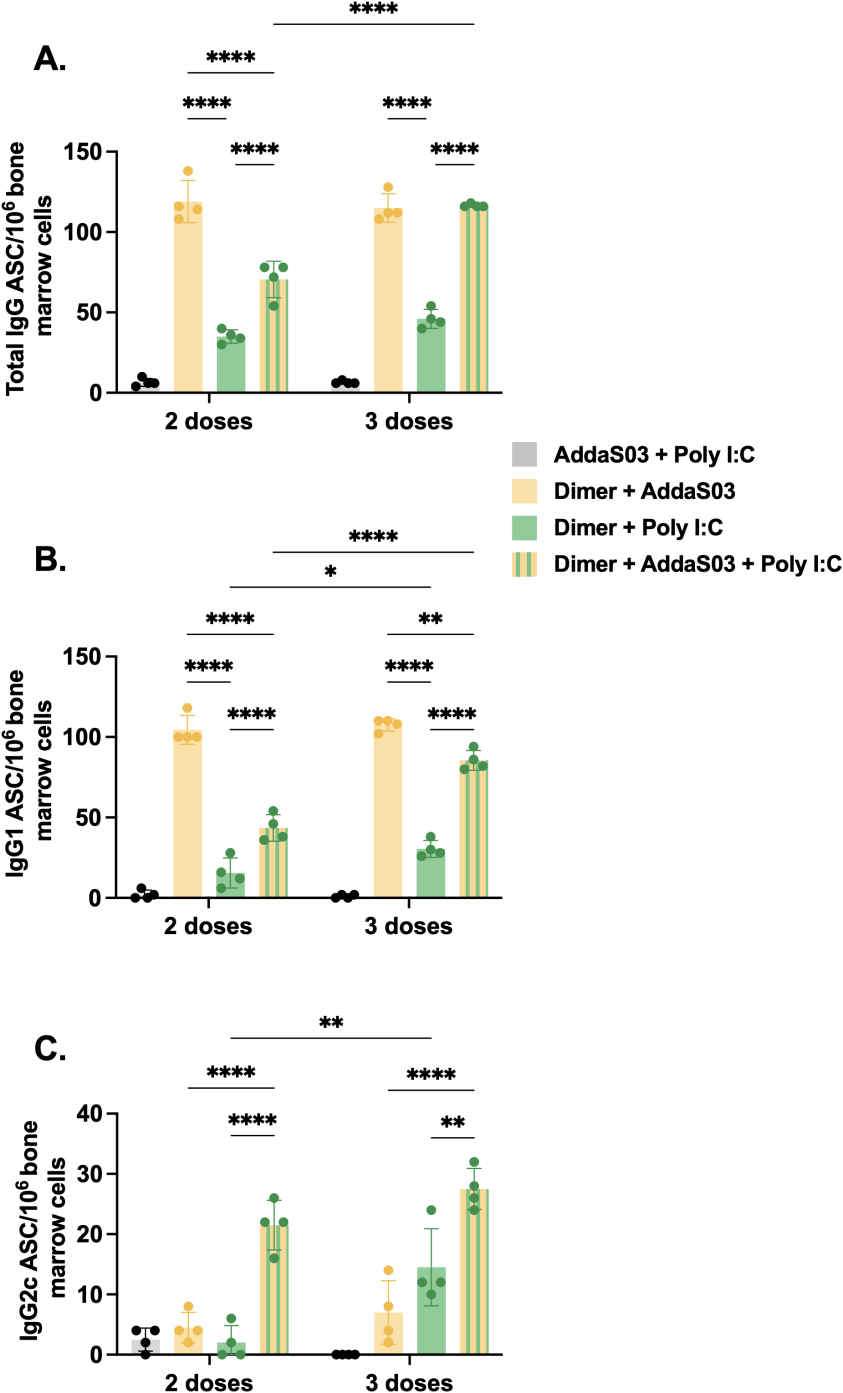
Bone marrow plasma cell frequency after two or three doses of dimer together with AddaS03, Poly I:C or AddaS03 + Poly I:C mixture. C57BL/6 mice were immunized subcutaneously with two or three doses of RBD dimer together with AddaS03, Poly I:C or AddaS03 + Poly I:C mixture. After the last dose, mice were euthanized, and bone marrow cells were cultured for 12 hours in dimer pre-coated plates. **(A)** Total IgG, **(B)** IgG1, and **(C)** IgG2c-producing bone marrow plasma cells were quantified by dimer-specific B cell ELISpot. Bone marrow cells were pooled and tested in quadruplicate. Data represent the mean ± SD. Statistical analysis was determined by two-way ANOVA followed by the Tukey *post-hoc* test. *p < 0.05, **p < 0.01, and ****p < 0.0001. ASC: antibody-secreting cells. Only statistically significant comparisons are depicted.

To further investigate the influence of adjuvants and dose-regimens on the frequency of long-lived bone marrow plasma cells, we measured the number of RBD-specific antibody-secreting cells (ASCs) by B cell ELISpot assay. ASCs refer to cells that migrated to bone marrow and are long-lived memory plasma cells which are known to secrete antibody for extended periods of time and maintain humoral immunity (36, 37). After two doses, the AddaS03 and AddaS03 + Poly I:C groups displayed the highest number of IgG-producing bone marrow plasma cells (**Figure 6A**). After the third dose, only the AddaS03 + Poly I:C group presented a substantial increase in ASC numbers. AddaS03 alone induced higher number of IgG1-producing bone marrow plasma cells than the other adjuvants, a pattern also observed after the third dose, although the difference was less pronounced due to an increase in the cell numbers in the AddaS03 + Poly I:C group (**Figure 6B**). After two doses, only the mixture of AddaS03 + Poly I:C induced IgG2c-producing plasma cells (**Figure 6C**). After the third dose, AddaS03 and Poly I:C alone increased the number of ASC, but both were still significantly inferior to the AddaS03 + Poly I:C mixture group (IgG2c 3 doses AddaS03 vs AddaS03 + Poly I:C, p < 0.0001; Poly I:C vs AddaS03 + Poly I:C, p < 0.01).

These data corroborate our previous findings of IgG subtypes in the serum, that the mixture of AddaS03 + Poly I:C efficiently induced quantitative and qualitative differences in antibody response.

### AddaS03 + Poly I:C performs similarly to Poly I:C and is superior to AddaS03 in inducing cellular responses

**Figure 7:**
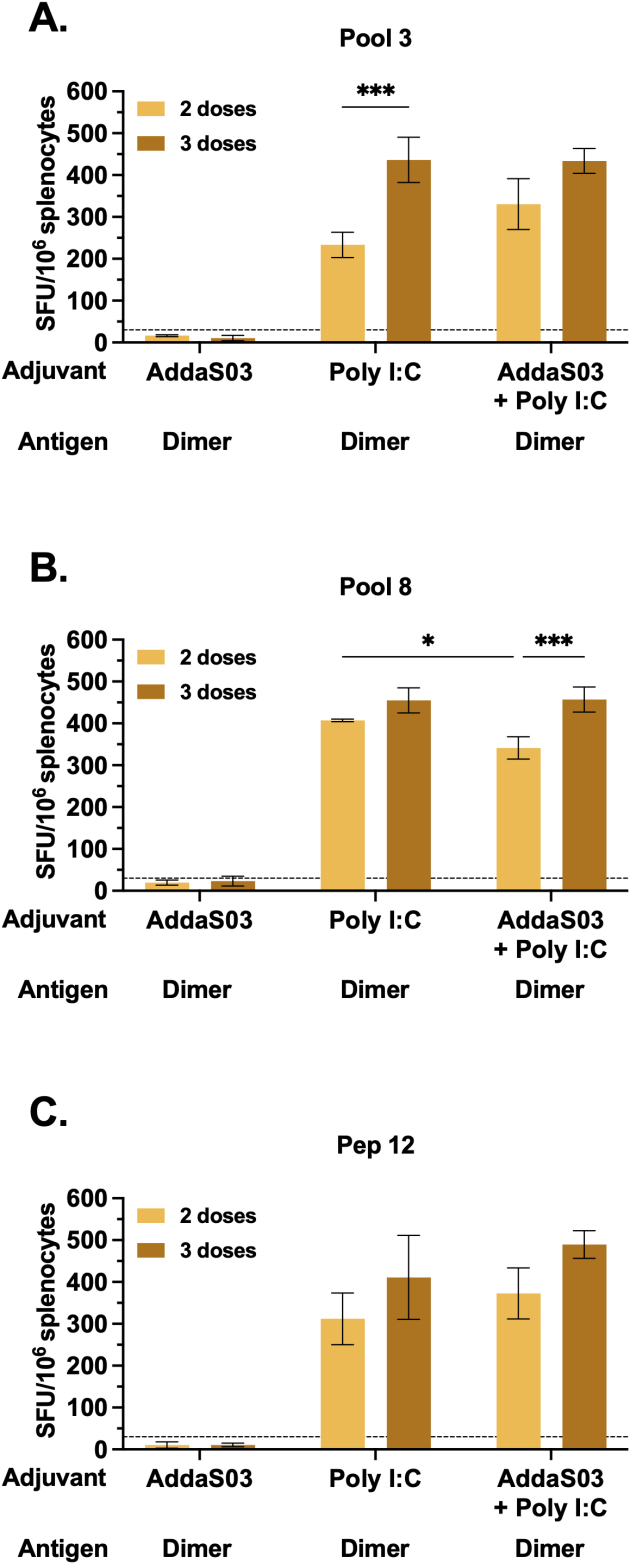
T cell response after two or three doses of RBD dimer together with AddaS03, Poly I:C or AddaS03 + Poly I:C mixture. C57BL/6 mice were immunized subcutaneously with two or three doses of RBD dimer, together with AddaS03, Poly I:C or AddaS03 + Poly I:C. After the last dose, splenocytes were harvested and cultured with dimer and peptides for 18 hours. Comparison of T cell responses after two or three-dose regimens against **(A)** pool 3, **(B)** pool 8, and **(C)** peptide 12. Cut-off=mean of control group + 3 SD. Splenocytes from each group were pooled and tested in triplicate. Data represent the mean ± SD. Statistical analysis was determined two-way ANOVA followed by the Tukey *post-hoc* test. *p < 0.05 and ***p < 0.001. Only statistically significant comparisons are depicted.

We next sought to investigate whether adjuvants induce specific cellular responses by IFNγ ELISpot. C57BL/6 mice were immunized as illustrated in **Figure 4A**, and splenocytes were harvested after the second or third doses. Mice that received dimer together with AddaS03 presented negligible numbers of IFNγ secreting cells, even after a third dose, whereas the Poly I:C and AddaS03 + Poly I:C groups performed better after two and three doses (**Supplementary Figures 4A, B**). Among the tested peptide pools, pools 3 and 8 were considered the top responders. In the Poly I:C group, the number of administered doses significantly influenced the magnitude of T cell responses measured against pool 3 (p < 0.001) (**Figure 7A**). Moreover, in the AddaS03+ Poly I:C group, there was a significant increased IFNγ response after the third dose against pool 8 (p < 0.001) (**Figure 7B**). After the third dose, no difference was observed between the Poly I:C and AddaS03+ Poly I:C adjuvanted groups.

Finally, we sought to map the potential individual peptides responsible for T cell activity. Using the DeconvoluteThis! software we selected potential peptides and among the suggestive combinations tested, peptide 12 (393-TNVYADSFVIRGDEVRQ-409) exhibited the highest magnitude of IFNγ response, with no difference between two and three doses (**Figure 7C**) in the Poly I:C and AddaS03+ Poly I:C adjuvanted groups.

These data demonstrated that the combination of AddaS03 with Poly I:C promoted a potent T cell response, similar to Poly I:C alone, which was directed mainly to pools 3 and 8, due to the presence of peptide 12. There was a slight increase in the magnitude of T cell response after the third dose. Taken together, our data suggest that a two-dose regimen of AddaS03 + Poly I:C is sufficient and optimal to stimulate potent humoral and T cell responses.

### Selected peptides encompass MHC class I and class II binders

**Figure 8:**
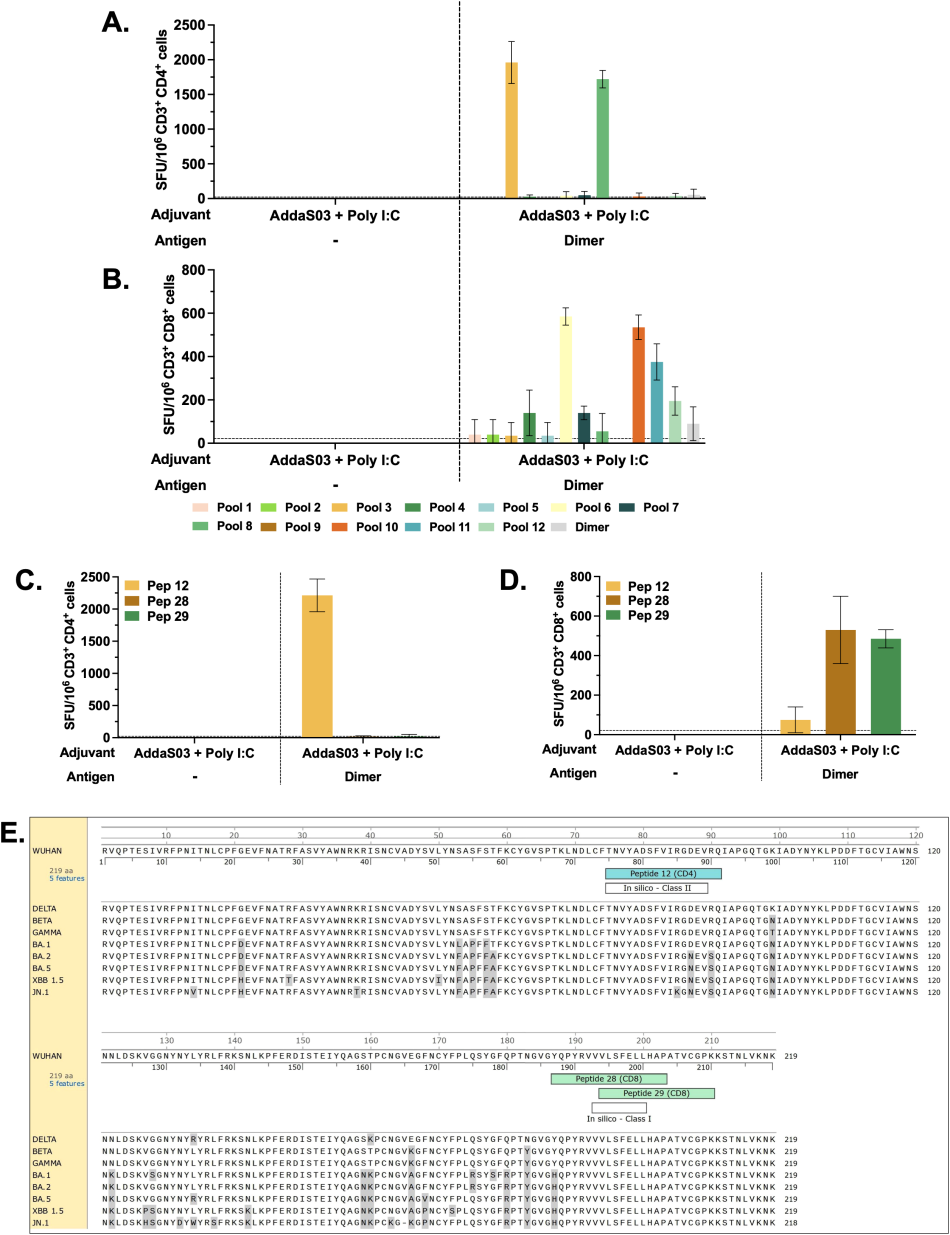
CD4^+^ and CD8^+^ T cell epitope mapping. Pooled spleens from groups that received three doses of dimer in the presence of AddaS03 + Poly I:C and control group (AddaS03 + Poly I:C) were sorted and utilized for **(A)** IFNγ ELISpot of CD4 and **(B)** CD8 T cells stimulated with peptide pools and RBD dimer protein. **(C)** IFNγ ELISpot of CD4^+^ and **(D)** CD8^+^ T cells stimulated with individual peptides (12, 28 and 29). Splenocytes from each group were pooled and tested in triplicate. Cut-off= mean of control group + 3 SD. Data represent the mean ± SD. **(E)** RBD amino acids sequence from Wuhan strain aligned with Delta, Beta, Gamma, Omicron BA.1, Omicron BA.2, Omicron BA.5, XBB 1.5 and JN.1. Amino acids highlighted in gray represent point mutations in variants compared to the Wuhan strain. *In silico* predictions using the IEDB MHC binding prediction tool. The top responders’ core peptides (highlighted in white) “in silico – Class II” (score = 0.5702) and “in silico – Class I” (score = 0.8929) aligned with the single peptides 12 (highlighted in blue), 28 (highlighted in green), and 29 (highlighted in green), respectively.

To identify class I and II -restricted epitopes, sorted T cells from mice that received dimer together with AddaS03 + Poly I:C and controls were utilized in IFNγ ELISpot. After three doses, IFNγ production by CD4^+^ T cells against pools 3 and 8 (**Figure 8A**) was detected only in mice that received the dimer with AddaS03 + Poly I:C. The CD8^+^ T cell response was directed against pools 6, 10 and 11, as well as against pools 4, 7 and 12, albeit with lower magnitude (**Figure 8B**). The DeconvoluteThis! analysis suggested peptides 12, 28 and 29 as potential epitopes derived from positive pools. When tested individually, peptide 12 was the main IFNγ inducer by CD4^+^ T cells (**Figure 8C**), whereas peptides 28 and 29 successively activated CD8^+^ T cells (**Figure 8D**).

Together, these data demonstrated that peptide 12 (393-TNVYADSFVIRGDEVRQ-409) is class II restricted, whereas peptides 28 (505-YQPYRVVVLSFELLHAP-521) and 29 (512-VLSFELLHAPATVCGPK-528) nest class I epitope. These results corroborate an *in silico* analysis using the IEDB MHC binding prediction tool (**Figure 8E**) that foresees the ability of a peptide to bind to specific MHC molecules. The top responders’ core peptides highlighted as “in silico – Class II” (score = 0.5702) and “in silico – Class I” (score = 0.8929) aligned with the single peptides 12, 28, and 29, respectively.

### Adjuvants influence RBD trimer- specific immune responses to a similar extent as dimer

To extend the observations about the impact of the combination of adjuvants and dose regimens, in a further series of experiments, we immunized mice with the same adjuvant formulations and the RBD trimer as an immunogen (**Supplementary Figure 5A**). Similarly to dimer immunization, AddaS03 and AddaS03 + Poly I:C induced higher titers of total IgG and IgG1 than Poly I:C (**Supplementary Figures 5B, C**). The AddaS03 + Poly I:C group also displayed significantly higher titers of IgG2c than the groups with AddaS03 or Poly I:C adjuvants (p < 0.0001 for both groups) (**Supplementary Figure 5D**). The affinity of antibodies generated after two doses with trimer in the presence of AddaS03+PolyI:C was also superior to that induced by the individual adjuvants and was sustained after the third dose (**Supplementary Figures 6A, B**). In PNA assays, AddaS03 + Poly I:C also performed similarly to AddaS03, being both superior to Poly I:C alone against Wuhan and Omicron pseudoviruses.

Furthermore, adjuvants also impacted T cell responses when RBD trimer was selected as immunogen (**Supplementary Figure 7**). Poly I:C and AddaS03 + Poly I:C also generated higher magnitude of IFNγ response than AddaS03 (**Supplementary Figure 7A**). Using bulk splenocytes, we observed an IFNγ response against peptides 12, 28, and 29 (**Supplementary Figure 7B**), corroborating our previous ELISpot results using sorted T cells.

Altogether, these data reinforce our observations with the dimer, showing that the phenomena are generated mainly by the mixture of adjuvants and dose regimens. Additionally, this dataset also supported the concept that the mixture of adjuvants generates a combination of the humoral response of AddaS03 with the Th1-skewing cellular capacity of Poly I:C.

## Discussion

Recent studies of vaccine candidates and COVID-19 patients have highlighted the potential of neutralizing antibodies and T cell response in controlling SARS-CoV-2 infection (38–41). Considering that adjuvants can impact not only the magnitude but also the quality of the immune response by influencing subclass switching, immunodominance and longevity, it is important to study how different adjuvants can modulate the response to recombinant proteins. Here, we compared the immunogenic properties of RBD monomer and dimer from the ancestral Wuhan strain and an RBD heterotrimer composed of Delta, Beta and Gamma VOCs. Furthermore, we screened different adjuvants to optimize both humoral and cellular responses.

Our findings demonstrated that all recombinant proteins elicited potent neutralizing activity against the ancestral strain both *in vitro* and *in vivo*. However, antibodies against RBD dimer and trimer, exhibited a broader neutralization profile against VOCs. Consistent with these findings, the favorable utilization of homodimer (20, 22, 23) and homotrimer (24, 25) has been previously observed, albeit with distinct multimerization strategies. In our model, where proteins were multimerized in tandem repeats, the homodimer effectively neutralized the Gamma and Delta variants; however, its activity against Omicron was notably reduced compared to the trimer. This observation aligns with findings reported during preclinical and phases I, II and III of Wuhan RBD homodimer-based vaccines (21, 42–44).

While the immunological benefits of RBD homomultimerization have been well documented (20–25), there is limited data on the immunogenicity of variant-based tandem antigens, particularly in trimeric conformations (45, 46). A broader neutralizing response against variants, reduced tissue viral loads and pathology and superior performance after challenge compared to homomultimer are common features observed in these studies. The RBD heterotrimer harbors mutations from the Delta (L452R, T478K), Beta (K417N, E484K, N501Y) and Gamma (K417T, E484K, N501Y) variants, which are also found in Omicron BA.1 and BA.2, with the exception of Delta’s L452R mutation (47). This likely explains the enhanced neutralization capacity of the trimer against the Omicron variant. Notably, antibodies generated against trimer not only effectively neutralized the variants included in its composition but also demonstrated substantial activity against the ancestral Wuhan strain. This may be attributed to the high similarity between the Delta RBD and the ancestral Wuhan RBD (99%), while the Beta and Gamma RBDs share a slightly lower similarity (98.6%). This is in line with previous work that demonstrated cross-neutralizing antibody response against ancestral Wuhan after immunization with a vaccine antigen containing four hot spot substitutions (48). Additionally, trimer immunization efficiently inhibited viral replication in the lungs and brain of animals challenged with the ancestral Wuhan strain, compared to the monomer. Therefore, the trimer was selected as the best immunogen in our study. The utilization of chimeric proteins as subunit vaccines could prove highly beneficial for effectively controlling multiple strains simultaneously, thereby enabling rapid formulation updates for future waves. However, direct comparisons between equimolar amounts of RBD heteromultimers and their respective homomultimer variants have yet to be performed.

In addition to the antigen, the nature of the adjuvant in a subunit protein-based vaccine impacts its immunogenicity and promotes the polarization of the immune system toward a beneficial axis. In the context of a virus infection such as SARS-CoV-2, the production of potent neutralizing antibodies is the most potent mechanism of vaccine efficacy (49, 50). We employed this rationale to initially screen between adjuvant compounds, evaluating their ability to induce binding and neutralizing antibodies. AddaS03 exhibited the highest overall capacity to induce antibody responses, corroborating previous observations (51).

AS03 has been successfully applied in humans for pandemic H1N1 (52, 53) and COVID-19 vaccines (54). Although the mechanism of action of AS03 is not fully understood, it induces high antibody titers and potent germinal center reactions (55), enhancing the persistence and clonal breadth of memory B cells (56). In our model, AddaS03 (AS03-like) induced high titers of IgG1 and less IgG2c but did not generate a potent IFNγ response, a phenomenon also described previously (57, 58). Interestingly, AddaS03 demonstrated overall superior efficacy compared to Addavax (similar to MF59), despite both being oil-in-water emulsion adjuvants. However, their compositions differ since Addavax consists of squalene emulsified in micelles containing polysorbate, whereas AddaS03/AS03 includes a combination of squalene and α-tocopherol, similarly emulsified in polysorbate (55). The addition of α-tocopherol seems to be crucial to its superior immunogenicity, as it promotes enhanced antigen uptake and dendritic cell maturation in the draining lymph nodes, when compared to Addavax (14). The mechanism underlying these events appears to involve the activation of the endoplasmic reticulum stress sensor IRE1α in monocytic cells. This activation triggers the unfolded protein response pathway, which induces cytokine production by innate immune cells, thereby amplifying the adjuvant properties of AS03 (59). Previous human studies also demonstrated that AS03 was superior to MF59 (similar to Addavax) in eliciting more potent and broader IgG responses (60–62).

A Th1-skewed cellular response is highly beneficial against viral pathogens and plays a critical role in the clearance of SARS-CoV-2 (41). To address this, we optimized our formulation by combining AddaS03 with Poly I:C, a classic Th1-inducing adjuvant, which led to a dramatic improvement in the magnitude of IFNγ response. This cytokine is also the major driver of IgG2c class switching in mice (63), and the increased induction of IFNγ by AddaS03 + Poly I:C mirrors the higher titers of this subclass. IgG2c interacts with all Fc receptors, exhibiting a higher binding capacity for activating receptors and is able to fix complement (63). The enhanced frequency of RBD-specific IgG2c-producing bone marrow plasma cells also points to the ability of this adjuvant combination to generate long-lived memory plasma cells as the migration of specific anti-RBD IgG2c-producing B cells to the bone marrow are indicative of the formation of long-lived bone marrow plasma cell populations (57). Thus, the mixture of adjuvants had synergistic effects, by combining the potent humoral ability of AddaS03 with the cellular Th1-skewing capacity of Poly I:C. In prior studies with RBD heterotrimers (45, 46), the constructs were administered using single adjuvant formulations such as Sepivac SWE or aluminum hydroxide, yielding mixed Th1-Th2 responses. Our study extends this knowledge by systematically evaluating the immunogenicity of a variant heterotrimer formulated with a combination of adjuvants (AddaS03 + Poly I:C).

The combination of adjuvants with distinct molecular mechanisms is widely accepted, and adjuvant systems are already implemented in vaccines against malaria, herpes zoster, human papillomavirus (HPV), and influenza (17–19, 64). The mixture of alum-3M-052 (TLR7/8 agonist) promoted a 100-fold higher neutralizing anti-RBD antibody titer compared to alum alone (24). Regarding the use of TLR agonists together with oil-in-water adjuvants, the combination of INI-2002 (a TLR4 agonist) and INI-4001 (a TLR7/8 agonist) with an AS03-like emulsion has been used with subunit RBD protein vaccines (65). The mixtures induced higher antigen-specific production of IFNγ and IgG2c titers, and a Th2 to Th1 shift after a two-dose regimen. The mixtures also broadened the neutralization against variants of concern (VOCs). These findings are consistent with our observations, highlighting the benefits of adding TLR agonists to AddaS03/AS03.

With regard to the dose regimen, our analysis indicated that a third dose enhanced antibodies’ affinity, while generating only slight increases in T and B cell responses. Thus, we concluded that a two-dose regimen of AddaS03 + Poly I:C is the most cost-effective scheme for generating both humoral and cellular responses.

Given that T-cell epitopes do not undergo many mutations between variants and that T cell responses are cross-reactive, the important role of T cell immunity in the prognosis of COVID-19 and the maintenance of immunity to SARS-CoV-2 (40, 41) underscore the importance of understanding T-cell responses induced after vaccination. SARS-CoV-2-specific T cells were previously associated with protection from symptomatic disease in vaccinated children (66). New vaccine candidates that are capable of inducing strong T-cell responses are currently being evaluated in clinical trials (67). Recent work has demonstrated that emerging variants that escape antibody-mediated neutralization are still recognized by cross-reactive T cells (68–70).

Peptide 12 (393-TNVYADSFVIRGDEVRQ-409) was class II restricted, and to the best of our knowledge, this is the first description of this peptide as an I-A/I-E class II epitope for C57BL/6 within the RBD sequence. This peptide also contains the core sequence VYADSFVIRG, which has been identified as a class I epitope restricted by H-2K_b_/D_b_ (71). In our study, the high neutralization capacity of antibodies generated by immunization with RBD proteins and the absence of anti-RBD IgG in CD4 KO mice point to the importance of peptide 12 as a cognate help. Peptides 28 (505-YQPYRVVVLSFELLHAP-521) and 29 (512-VLSFELLHAPATVCGPK-528) also elicited a T cell response, albeit to a lesser magnitude. Their sequences share a core VLSFELL sequence, previously demonstrated by distinct groups (72, 73) to be a H-2K^b^ restricted epitope, corroborating our data. Here, we showed that the immunodominant peptides from the ancestral Wuhan strain were highly conserved among Delta, Beta and Gamma VOCs, present in our RBD heterotrimer construct, as well as in Omicron BA.1. However, compared to Omicron BA.2, BA.5, XBB 1.5 and JN.1, there are two substitutions within the class II peptide 12: D405N and R408S. Additionally, variant JN.1 exhibits an additional mutation, R403K. These mutations may influence the T cell response to these variants as they reside in the class II peptide 12 described, although further experiments are required to validate this hypothesis. The JN.1-derived variants KP.3.1.1, XEC, and KP.2.3 present the cited mutations as well as S31del and T22N, located in the N-terminal domain (NTD) of the spike protein which contribute to a significant reduction in neutralizing antibody responses compared to the parental strain (74, 75). The direct impact of these mutations in the context of RBD vaccination may be minimal. Additionally, these variants possess two mutations in the RBD—F456L and Q493E—though neither of these mutations falls within the T cell epitopes described in our study. The identification of RBD-derived MHC class I and class II peptides in C57BL/6 mice - the genetic background of the widely used, SARS-CoV2 susceptible K18-hACE2 transgenic model- is of utmost importance. To our knowledge, this is the first study to report both CD4^+^ and CD8^+^ T cell-restricted epitopes within the RBD for this mouse strain, providing a valuable reference for future preclinical vaccine studies. One limitation of our study is that we did not directly compare the Delta, Beta and Gamma RBD heterotrimer with an ancestral Wuhan RBD homotrimer construct, which would be important to further explore the mechanism of the broader neutralization capacity of this protein. A head-to-head comparison of Delta, Beta and Gamma RBD monomers to heterotrimer RBD protein would also be fruitful for understanding the role of protein assembly conformation in the dynamics of the immune response against the VOCs. In addition, whether immunization with Wuhan dimer construct could protect against challenge with Beta, Gamma and Omicron VOCs remains an open question. Furthermore, we did not perform analysis of the T cell responses against the class II peptide 12 harboring D405N and R408S mutations observed in Omicron BA.2, BA.5, XBB 1.5 and JN.1. Overall, our findings provide insight into how immunogen design and adjuvant formulation influence immunity, which is of utmost importance for preparedness against emerging threats. This could provide valuable insights for the design of future vaccines that can more effectively target evolving variants of SARS-CoV-2 or other coronaviruses of pandemic potential.

## Data Availability

The raw data supporting the conclusions of this article will be made available by the authors, without undue reservation.
