# Supplementary material for: Adjuvant combination and antigen multimerization shape neutralizing antibody and T cell responses to a SARS-CoV-2 RBD subunit vaccine
Source: Front Immunol. 2025 Jul 17;16:1610422. doi: 10.3389/fimmu.2025.1610422 (PMC12310705; doi:10.3389/fimmu.2025.1610422)
Supplement: Supplementary file 1 [file DataSheet1.docx]

**Supplementary Figures**

**Adjuvant combination and antigen multimerization shape neutralizing antibody and T cell responses to a SARS-CoV-2 RBD subunit vaccine**

João Pedro da Silva Nunes, Mariângela de Oliveira Silva, Juliana de Souza Apostolico, Isabela Pazotti Daher, Rodolfo Ferreira Marques, Marcio Massao Yamamoto, Alexia Adrianne Venceslau Brito Carvalho, Maria Fernanda de Castro Amarante, Edison Luiz Durigon, Carsten Wrenger, Luiz Mario Ramos Janini, Edmarcia Elisa de Souza, Robert Andreata-Santos, Juliana Terzi Maricato, Edecio Cunha- Neto, Jorge Kalil, Silvia Beatriz Boscardin, Daniela Santoro Rosa

**Supplementary Figure 1**

*
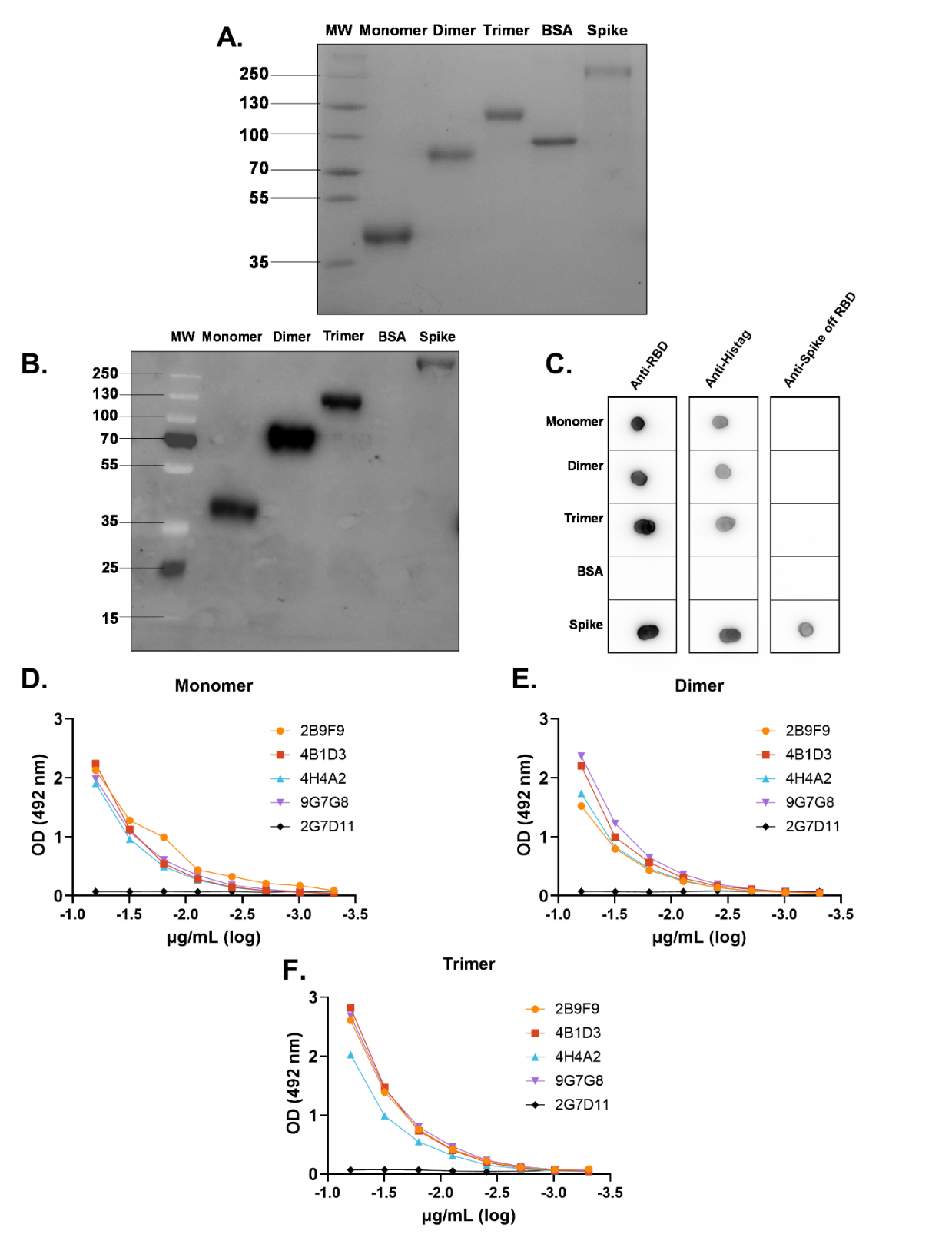
*

**Supplementary Figure 1: Protein characterization and immunorecognition.** **(A)** SDS-PAGE (12%) of recombinant monomer, dimer and trimer. **(B)** Western blot with recombinant proteins and anti-RBD pooled monoclonal antibodies (2B9F9, 4H4A2, 4B1D3, 9G7G8). **(C)** Dot Blot of native recombinant proteins with anti-RBD pooled mAbs (2B9F9, 4H4A2, 4B1D3, 9G7G8), anti-6x-HisTag and anti-Spike mAb off-RBD site (2G7D11). **(D)** Absorbance curves (492nm) with monomer, **(E)** dimer and **(F)** trimer- coated plates with different concentrations of the mAbs 2B9F9, 4B1D3, 4H4A2, 9G7G8 and 2G7D11.

**Supplementary Figure 2**

**
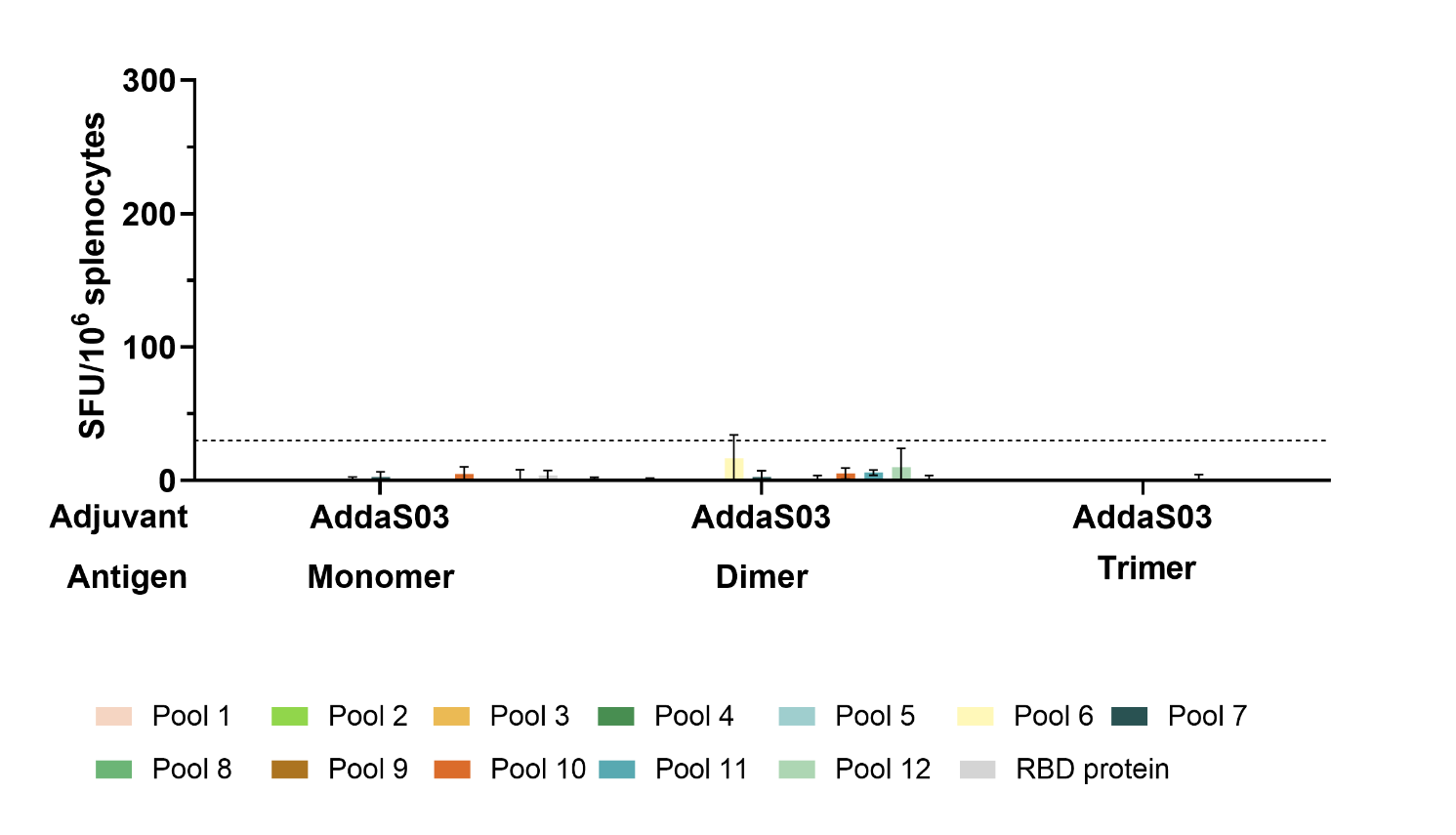
Supplementary Figure 2: T cell analysis after two doses of RBD monomer, dimer or trimer together with AddaS03.** C57BL/6 mice were immunized subcutaneously with two doses of RBD monomer, dimer or trimer together with AddaS03. For IFN-γ T cell ELISpot, splenocytes were harvested after the last dose and cultured with respective RBD protein or peptide pools for 18 hours. Cut-off = mean of control group + 3 SD. Splenocytes from each group were pooled and tested in triplicate. Data represent the mean ± SD. Statistical analysis was determined by two-way ANOVA followed by Tukey post-hoc test. Only statistically significant comparisons are depicted.

**Supplementary Figure 3**

*
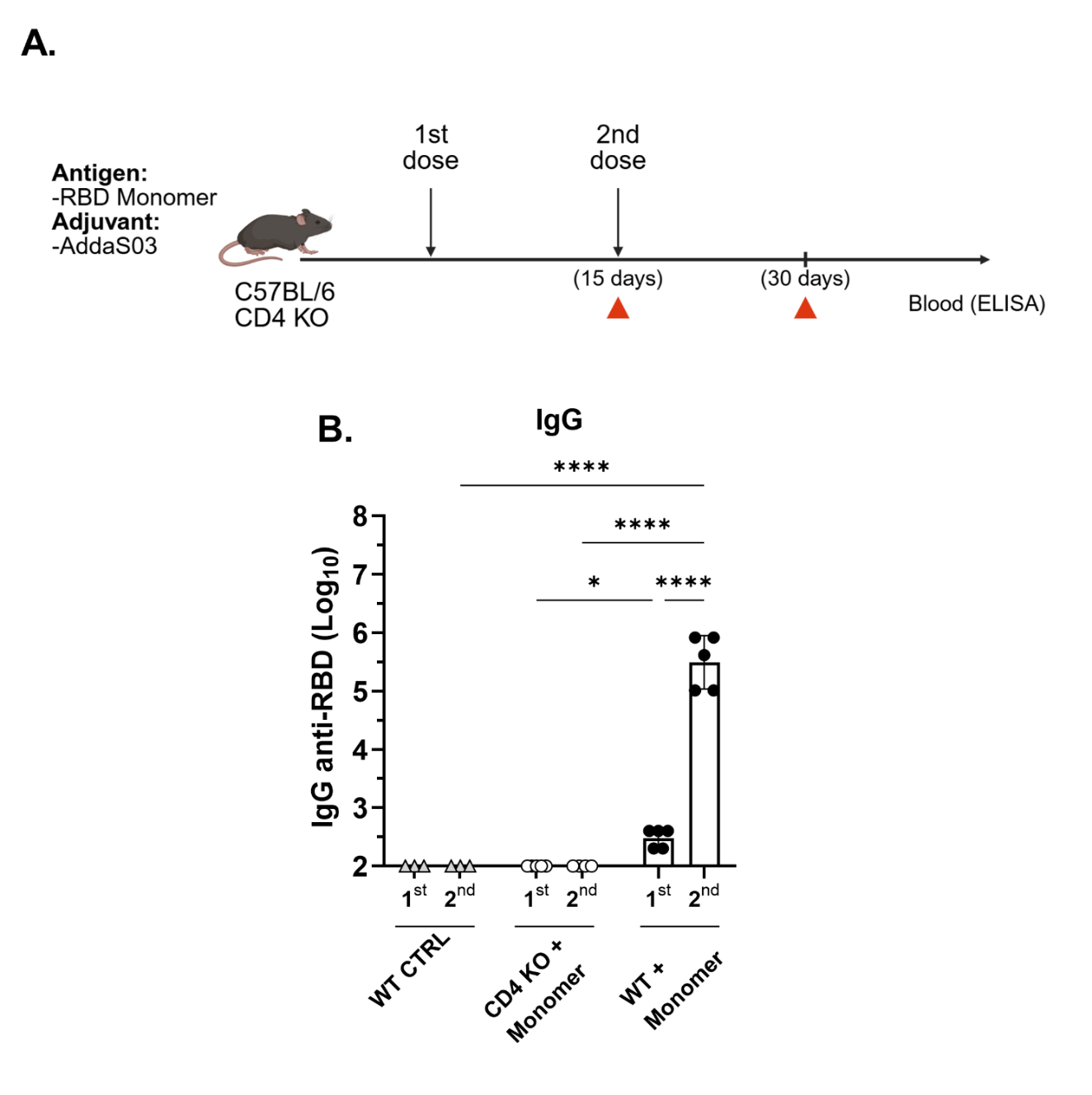
*

**Supplementary Figure 3: CD4 KO mice do not produce specific anti-RBD after immunization.** **(A)** Study design. C57BL/6 and CD4 KO mice (n=5) were immunized subcutaneously with two doses of RBD monomer together with AddaS03 or with adjuvant only (CTRL, n=3). **(B)** Anti-RBD total IgG titers 15 days after each dose. Serum of each animal was assayed individually. Data represent the mean ± SD. Statistical analysis was determined by two-way ANOVA followed by Tukey post-hoc test. * p < 0.05, and **** p < 0.0001. Only statistically significant comparisons are depicted.

**Supplementary Figure 4**


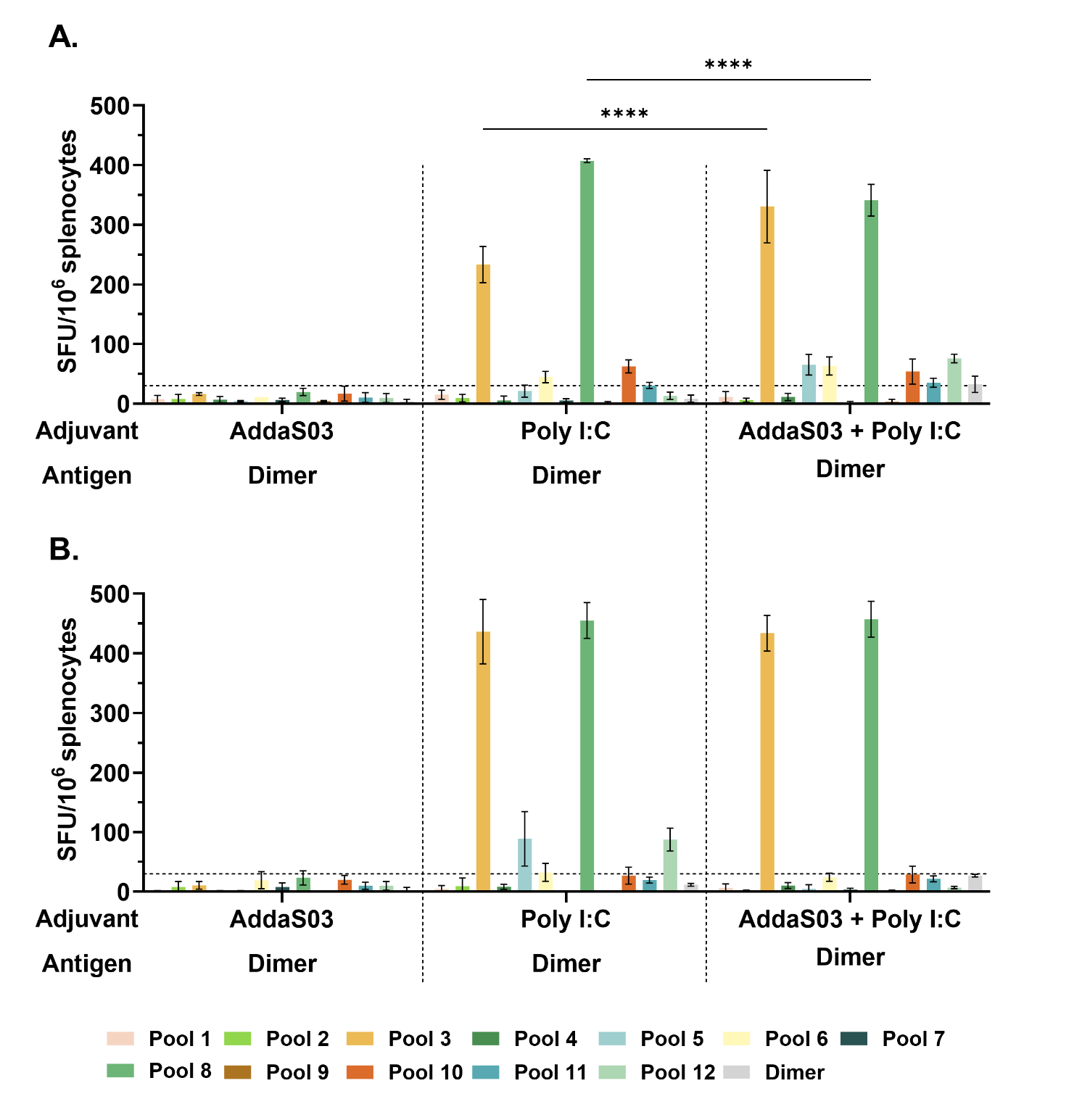


**Supplementary Figure 4: T cell analysis after two or three doses of RBD dimer together with AddaS03, Poly I:C or AddaS03 + Poly I:C.** C57BL/6 mice were immunized subcutaneously with two or three doses of RBD dimer together with AddaS03, Poly I:C or AddaS03 + Poly I:C mixture. After the last dose, splenocytes were harvested and cultured with RBD dimer or peptide pools for 18 hours. IFN-γ T cell ELISpot after **(A)** two and **(B)** three doses. Cut-off= mean of control group + 3 SD. Splenocytes from each group were pooled and tested in triplicate. Data represent the mean ± SD. Statistical analysis was determined by two-way ANOVA followed by Tukey post-hoc test. **** p < 0.0001. Only statistically significant comparisons are depicted.

**Supplementary Figure 5**

***
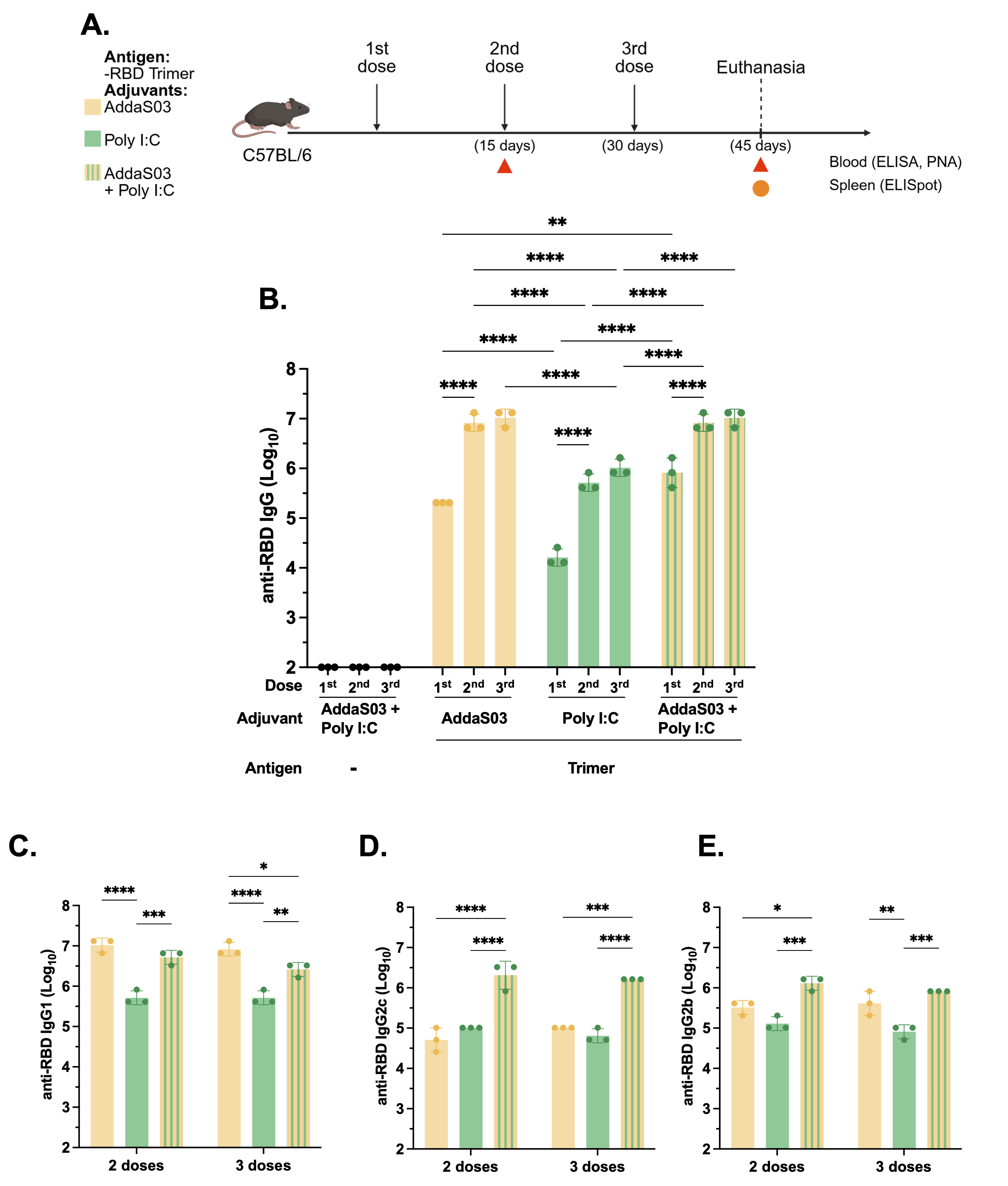
***

**Supplementary Figure 5: Humoral analysis after two or three doses of trimer with AddaS03, Poly I:C or AddaS03 + Poly I: C mixture. (A)** Study design. C57BL/6 mice (n=3) were immunized subcutaneously with two or three doses of RBD trimer together with AddaS03, Poly I:C or AddaS03 + Poly I:C mixture. **(B)** Total anti-RBD IgG titers 15 days after each dose. **(C)** Anti-RBD IgG1, **(D)** IgG2c and **(E)** IgG2b subtypes after two or three doses. For ELISA and PNA, serum of each animal was assayed individually. Data represent the mean ± SD. Statistical analysis was determined by two-way ANOVA followed by Tukey post-hoc test. * p < 0.05, ** p < 0.01, *** p < 0.001, and **** p <0.0001. Only statistically significant comparisons are depicted.

**Supplementary Figure 6**

**
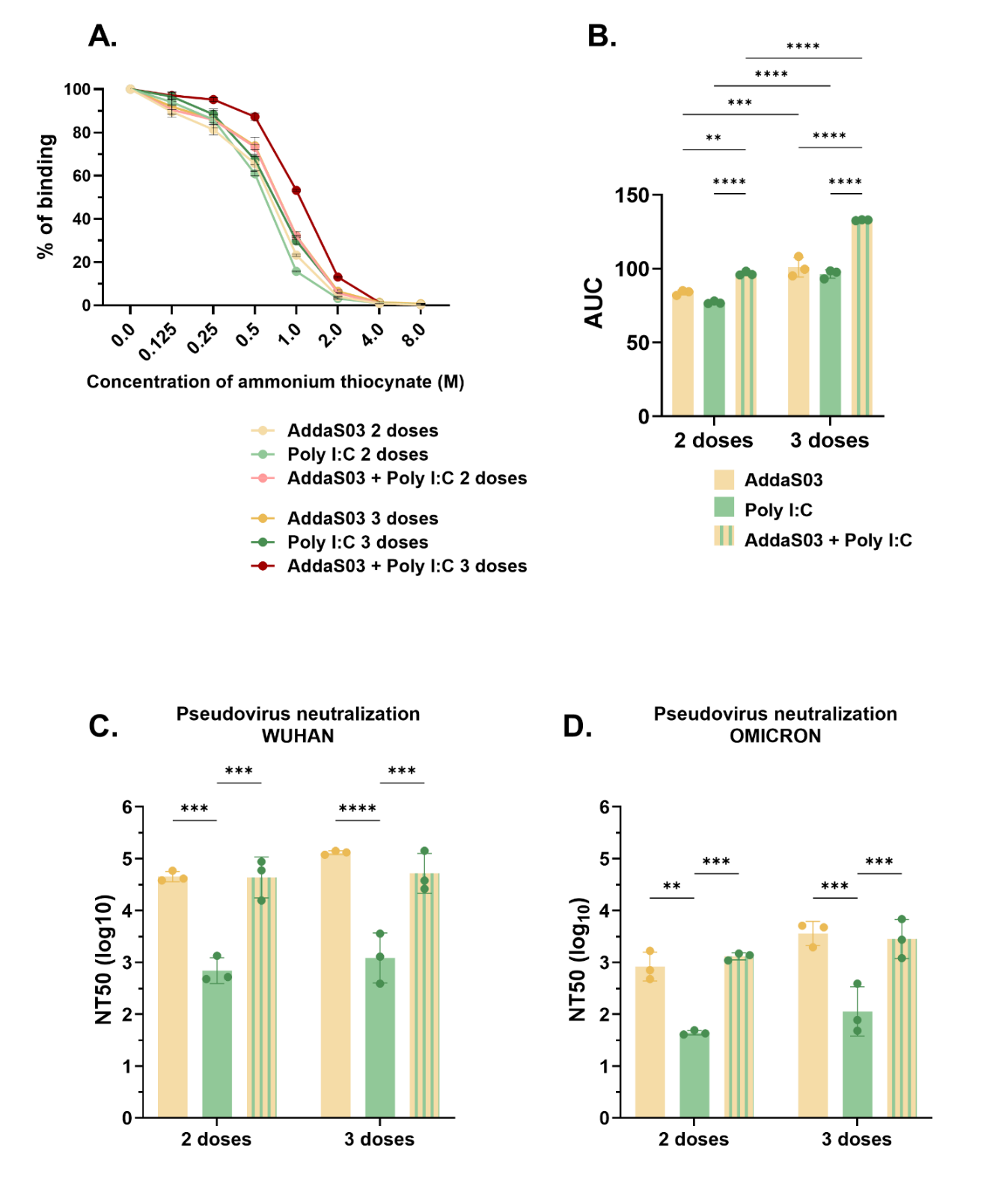
**

**Supplementary Figure 6: Affinity and neutralization after two or three doses of RBD trimer together with AddaS03, Poly I:C or AddaS03 + Poly I: C mixture.** C57BL/6 mice were immunized subcutaneously with two or three doses of RBD dimer together with AddaS03, Poly I:C or AddaS03 + Poly I:C mixture. **(A)** Antibody affinity of pooled mouse sera after incubation with increasing concentrations of ammonium thiocyanate. **(B)** Area under the curve of affinity assay. **(C)** NT50 PNA against Wuhan and **(D)** Omicron BA.2 15 days after two or three doses. For PNA, serum of each animal was assayed individually. For affinity, serum of each group was pooled and tested in triplicate. Data represent the mean ± SD. Statistical analysis was determined by two-way ANOVA followed by Tukey post-hoc test. ** p < 0.01, *** p < 0.001, and **** p < 0.0001. Only statistically significant comparisons are depicted.

**Supplementary Figure 7
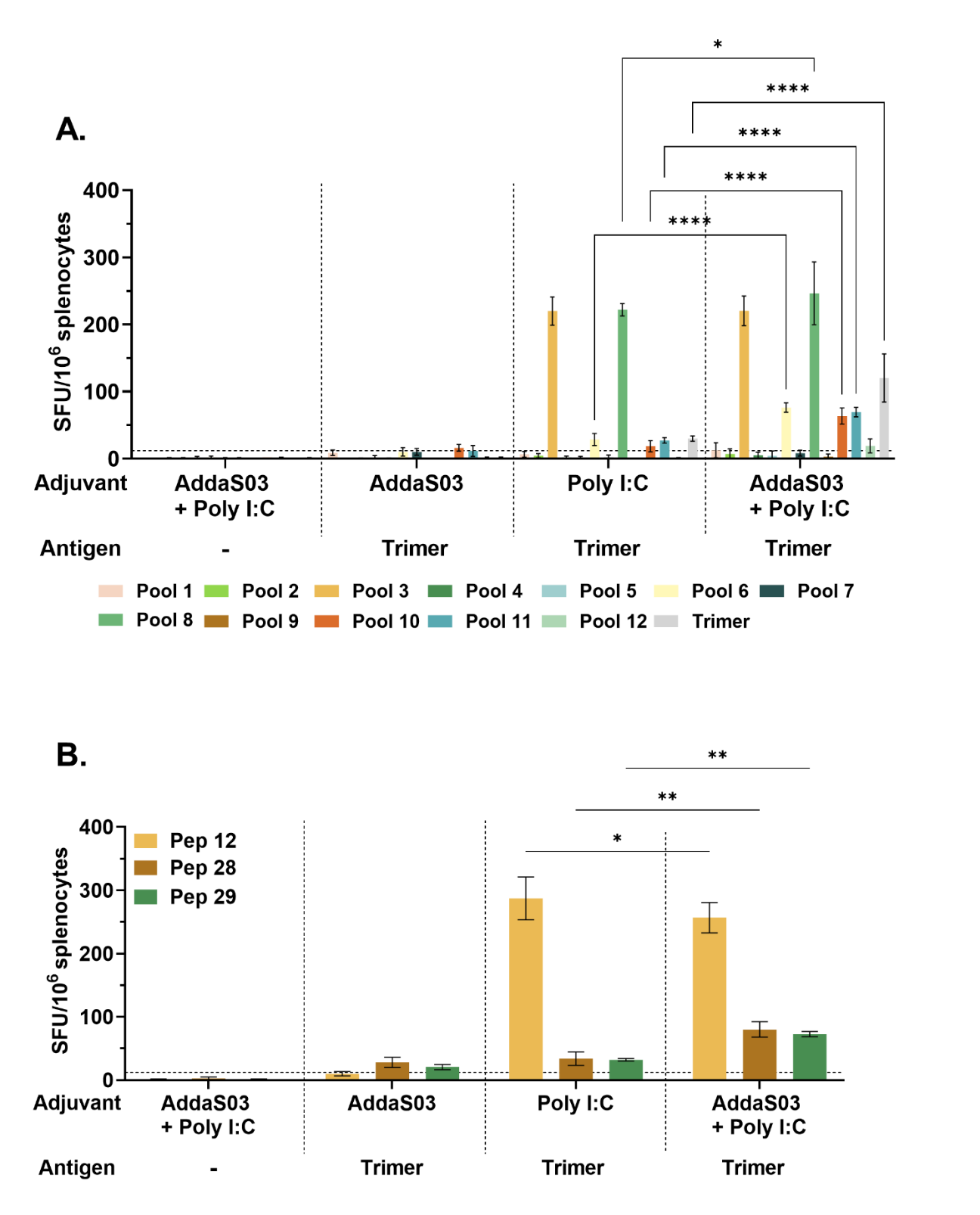
Supplementary Figure 7: T cell response after three doses of RBD trimer together with AddaS03, Poly I:C or AddaS03 + Poly I:C.**C57BL/6 mice were immunized subcutaneously with three doses of RBD trimer together with AddaS03, Poly I:C or AddaS03 + Poly I:C mixture. After the last dose, bulk splenocytes were harvested and cultured with peptides and recombinant RBD trimer for 18 hours. IFN-γ T cell ELISpot against **(A)** peptide pools and **(B)** peptides 12, 28 and 29. Splenocytes from each group were pooled and tested in triplicate. Cut-off= mean of control group + 3 SD. Data represent the mean ± SD. Statistical analysis was determined by two-way ANOVA followed by Tukey post-hoc test. * p<0.05, ** p < 0.01, and **** p < 0.0001. Only statistically significant comparisons are depicted.
